# Exploring Fold Space Preferences of New-born and Ancient Protein Superfamilies

**DOI:** 10.1371/journal.pcbi.1003325

**Published:** 2013-11-14

**Authors:** Hannah Edwards, Sanne Abeln, Charlotte M. Deane

**Affiliations:** 1 Department of Statistics, University of Oxford, Oxford, United Kingdom; 2 Department of Computer Science, Vrije Universiteit, Amsterdam, The Netherlands; University College London, United Kingdom

## Abstract

The evolution of proteins is one of the fundamental processes that has delivered the diversity and complexity of life we see around ourselves today. While we tend to define protein evolution in terms of sequence level mutations, insertions and deletions, it is hard to translate these processes to a more complete picture incorporating a polypeptide's structure and function. By considering how protein structures change over time we can gain an entirely new appreciation of their long-term evolutionary dynamics. In this work we seek to identify how populations of proteins at different stages of evolution explore their possible structure space. We use an annotation of superfamily age to this space and explore the relationship between these ages and a diverse set of properties pertaining to a superfamily's sequence, structure and function. We note several marked differences between the populations of newly evolved and ancient structures, such as in their length distributions, secondary structure content and tertiary packing arrangements. In particular, many of these differences suggest a less elaborate structure for newly evolved superfamilies when compared with their ancient counterparts. We show that the structural preferences we report are not a residual effect of a more fundamental relationship with function. Furthermore, we demonstrate the robustness of our results, using significant variation in the algorithm used to estimate the ages. We present these age estimates as a useful tool to analyse protein populations. In particularly, we apply this in a comparison of domains containing greek key or jelly roll motifs.

## Author Summary

Proteins are the molecular workers of the cell. They are formed from a string of amino acids which folds into an elaborate three-dimensional structure. While there is a relationship between a protein's sequence and its structure this relationship is highly complex and not fully understood. Protein structures tend to evolve differently to their sequences. They are far more conserved so tend to change slower. The aim of this paper was to identify trends in the way that protein structures evolve, rather than adapting models of sequence evolution. To do this we have provided a database of ages for structural superfamilies. These ages are robust to drastic differences in the evolutionary assumptions underlying their estimation and can be used to study differences between populations of proteins. For example, we have compared newly evolved structures against those with a long evolutionary history and found that, overall, a shorter evolutionary history corresponds to a less elaborate structure. We have also demonstrated here how these ages can be used to compare particular structural motifs present in a large number of protein structures and have shown that the jelly roll motif is significantly younger than the greek key.

## Introduction

The current wealth of freely available genetic sequences offers the potential to uncover the evolutionary history of genes and their products, proteins. While there exist no remains of primitive proteins, extant protein information can be used to estimate a protein family's history. This approach is particularly well suited to structural information. Protein structures are far more conserved than their sequences and thus preserve a deep phylogenetic signal [1]. Furthermore, for the majority of globular proteins, a stable three-dimensional structure is thought to be a requirement for many aspects of its function. By maintaining the precise positioning of functional residues while also minimising other undesirable interactions a protein's structure is intimately linked to the role it plays within the cell [2]. Moreover, phylogenetic trees built using the structural content of species' proteomes have been shown to produce more reliable topologies than trees constructed using their protein sequences [3]. These observations support the use of structure as a fundamental molecular unit when studying the evolution of proteins. Furthermore, they suggest that any conversation on the evolution of proteins must first understand the major driving forces behind such changes from a structural perspective.

In order to visualise the landscape and diversity of structure space protein structures have been clustered within a hierarchical taxonomy [4], [5]. The SCOP database is one such manual classification scheme which, at the superfamily level, attempts to cluster together protein domains with a common evolutionary origin, based primarily on strong functional and structural similarity [6]. The superfamily classification lies in between the family level, largely defined by a domain's amino acid sequence, and the fold, a structural consensus of a domain's topology. In this work we primarily consider sets of structural domains classified as superfamilies in SCOP 1.75.

Despite the potential for rich diversity within the structural universe it is surprising how sparse this space remains [7]. The current repertoire of proteins with known structure fall into less than 1,200 unique SCOP folds and the majority of these contain only one sequence family [8]. While this is unlikely to represent the true diversity of naturally occurring proteins and current projections for the size of protein fold space range from around 2,000 [9] to over 10,000 [10], it is thought that the vast majority of extant proteins will fall into only around 1,000 common folds [11]. Furthermore, the landscape of this core fold space is highly heterogeneous, with a few so called ‘superfolds’ densely populated by sequence families [12]. The unique composition of this space is a consequence of protein evolution through neutral drift and active selection together with a complex interplay of other factors such as genome structure, mutational mechanisms, function and the need for interactions, all of which close off portions of the configuration space. However, little is known about the exact nature by which the range of protein structures we see today have evolved [2].

One way in which we can seek to explore the forces behind such a history is to consider annotating the protein structure universe with an estimate of its evolutionary age [7], [13], [14]. The age of a population of proteins is the estimated node age of its first ancestor across a phylogeny of completely sequenced genomes. This method has been implemented for both structural superfamilies [13], [15] and sequence families [7], [14], although the latter tend to involve a much reduced phylogenetic tree and evolutionary scale. Methods for predicting the internal node of the ancestor for a given family or superfamily also vary. A maximum parsimony model for superfamily evolution has been largely adopted for this step [13], [15], [16], although alternatives include Dollo parsimony: taking the most recent common ancestor [7], [13].

These parsimony models take as input a phylogenetic species tree and the occurrence profile of each structural superfamily across this set of species. The occurrence profile for a superfamily is simply its presence or absence on each of the genomes [16]. Parsimony attempts to reconstruct the most likely series of gain and loss events at internal nodes of the tree which explain the occurrence profile at its leaves. The likelihood of these events is based on simple assumptions relating to the evolution of protein domains. The principle underlying all types of parsimony is that the scenario of events involving the least evolutionary change is preferred. Gain events can represent de novo superfamily gain, lateral gene transfer of a superfamily between genomes, and a false positive assignment of a superfamily to a genome. Loss events can represent the loss of a superfamily and also false negative assignments to a genome. Maximum parsimony methods allow for a weighting of the likelihood of loss events relative to gain events, while Dollo parsimony considers a gain event to be so rare it is most likely to have occurred only once in the evolution of a superfamily. Since lateral gene transfer is rare between Eukaryotes but may be quite common among Prokaryotes it has been suggested that maximum parsimony is an appropriate model for Prokaryotic genomes while Dollo parsimony should be used for Eukaryotes [16], [17].

Previous studies have shown a significant positive correlation between the age of a domain's structure and its length [7], [14]. These results remain pronounced over different methods for calculating the age of a superfamily or protein sequence. This seemingly fundamental relationship between the age of a structure and its length has supported the idea that the primitive protein universe was populated mainly by small folds [7]. In fact, the recent success in using structural fragments to predict protein structures (see, for example [18]) has further stimulated debate as to whether the evolutionary origins of the current fold space are in fact short peptide fragments that have combined to form larger folds [19].

It has also been reported that 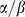 class domains tend to be significantly older than superfamilies belonging to other classes [13]. 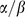 domains also tend to be significantly longer than other classes but they are also distinguishable in other respects [20]. They are unique among the classes in containing a majority of parallel 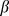-strands as opposed to the antiparallel structure which characterise all-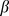 and 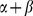 classes. 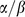 folds also contain a large number of the so-called ‘superfolds’: folds containing large numbers of different superfamilies and a high proportion of all determined structures [12]. Such 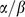 superfolds include P-loop NTPases, Rossmann folds and TIM barrels [11].

In this work we present phylogenetic profiles and evolutionary ages for superfamilies representing the current known structural universe. We show that these age estimates are largely robust to different evolutionary models, datasets and phylogenetic trees.

We compare the structural characteristics of two protein populations: new-borns, with biologically recent structural ancestors, and ancients, with ancestors at the root of the tree of life. Our results identify several characteristics that differ between the two populations. These differences support known relationships, such as the propensity of 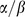 and longer superfamilies to be ancient, and also postulate several previously unseen characteristics which correlate with age.

While these structural relationships are marked we considered the possibility that they were the result of an asymmetry in the functional annotation of fold space. Here we show that our structural partitions result in far more dramatic age differences than functional groupings and as such the relationships between structure and age are not a residual effect of functional preferences.

## Results

**Figure 1:**
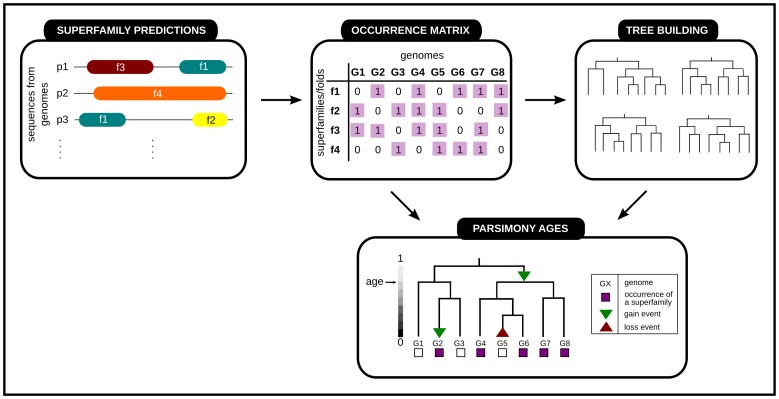
What do we mean by the age of a superfamily? Ages are generated using a phylogenetic species tree and an occurrence profile of a superfamily across the genomes of these species. Parsimony algorithms predict the simplest scenario of loss and gain events on internal nodes of the tree which explain the occurrence profile at its leaves. Ages are normalised between 0, at the leaves of the tree, and 1, at its root. Ancient superfamilies are predicted an age of 1 and new-born superfamilies are estimated to have an evolutionary age 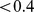.

1,847 SCOP superfamilies are annotated with an estimate of their age relative to a tree of life incorporating 1,014 completely sequenced genomes across the three superkingdoms (Archaea, Bacteria and Eukarya). These ages can be found online at http://www.stats.ox.ac.uk/research/proteins/resources. The superfamily age is a relative measure of when that superfamily first appeared, calculated according to parsimonious interpretations of evolutionary events. Figure 1 gives an outline of the age estimation procedure. These ages are used to discriminate the set of superfamilies into different age groups. There are 557 ancient superfamilies, that are predicted to have first evolved at the root of the tree (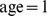) and 443 new-born superfamilies, predicted to have an ancestor nearer the leaves of the tree (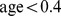). As there is not a single standard tree of life we calculate age estimates using 8 different phylogenetic trees (see methods for descriptions of the different trees).

### Robustness of superfamily ages and preferences

Superfamily ages are sensitive to the phylogenetic tree of life used, the prediction of superfamilies on genome sequences for the occurrence profiles, and to the parsimony method and parameters used to estimate events. In order to investigate the robustness of our age estimates to these assumptions we undertook our analysis across several phylogenetic trees and multiple parsimony models. We also explored the effect on our results of using different datasets: changing both the occurrence profiles and the set of genomes considered.

#### Parsimony method

In this work we have primarily used a maximum parsimony algorithm to estimate superfamily ages. One of the most significant assumptions within the maximum parsimony model is the ratio of the probability of a loss event relative to a gain event [21]. There is, to our knowledge, no comprehensive assessment of the biological relevance for different values of this parameter for structural superfamily evolution. The results we present here follow previous studies in assuming that these two events are equally likely to occur at any internal node [13], [16]. However we also predicted age estimates using a range of values for this parameter, up to a ten-fold asymmetry in the relative likelihood of both gain and loss events. As expected, the age estimates were sensitive to the change in this parameter, although they still maintained a strong correlation to ages calculated with a relative gain weight of 1 (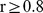). Moreover, the fold space preferences we report were upheld under the variation of this parameter. The results of this analysis for SCOP class, strand direction and domain length are given in the Figure S1.

As we mentioned in the Introduction, gain events in the tree represent gene gain but also false positives in the occurrence data as well as lateral gene transfer events. Since lateral gene transfer rarely occurs among Eukaryotic genomes it is perhaps more biologically relevant to consider the weights placed on gain events differently when considering the Eukaryotic tree of life [17]. We therefore also calculated ages using a fusion parsimony method: assigning events based on Dollo parsimony within the Eukaryotic subtree and according to maximum parsimony at the root and within the Bacterial and Archaeal subtrees. These fusion ages are strongly correlated to those estimated using the maximum parsimony model on the entire tree (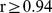 over equivalent phylogenetic trees) and, moreover, support the fold space preferences we report in the main body of the Results (see Figure S2). For simplicity, we have reported our results using the maximum parsimony ages, although the ages calculated using the fusion model, as well as those estimated using different gain weights, are also available to download.

#### Phylogenetic trees

For each method we also estimated ages across 8 different phylogenetic trees, including the NCBI common taxonomy tree. The ages generated using these different topologies were strongly correlated (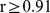 under a maximum parsimony model). Any result described here is significant using ages from any of these trees.

#### Other datasets

Ages calculated using data from SUPERFAMILY from October 2011 were strongly correlated (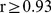 over equivalent phylogenetic trees) to the estimates presented here on the newer data. More significantly, ages calculated using an earlier version of SCOP (1.65) with reduced coverage on a much smaller set of genomes also supported the fold space preferences for new-born and ancient superfamilies which we report here.

SCOP superfamilies were chosen as the unit of this analysis because they are thought to represent definitive evolutionary relationships. They remain, however, a manually classified construct. To avoid any bias in their assignment we also performed the same analysis ages calculated at the fold level of the SCOP hierarchy. Using these fold ages produced the same results regarding properties of new-born and ancient folds as were seen using the superfamily ages.

### Structural Preferences

Representative domains for these superfamilies were taken from the ASTRAL database [22]. A number of different properties pertaining to the sequences, structures and functions of these domains were then used to compare the ancient and new-born populations.

#### Secondary structure: SCOP class and strand direction

**Figure 2:**
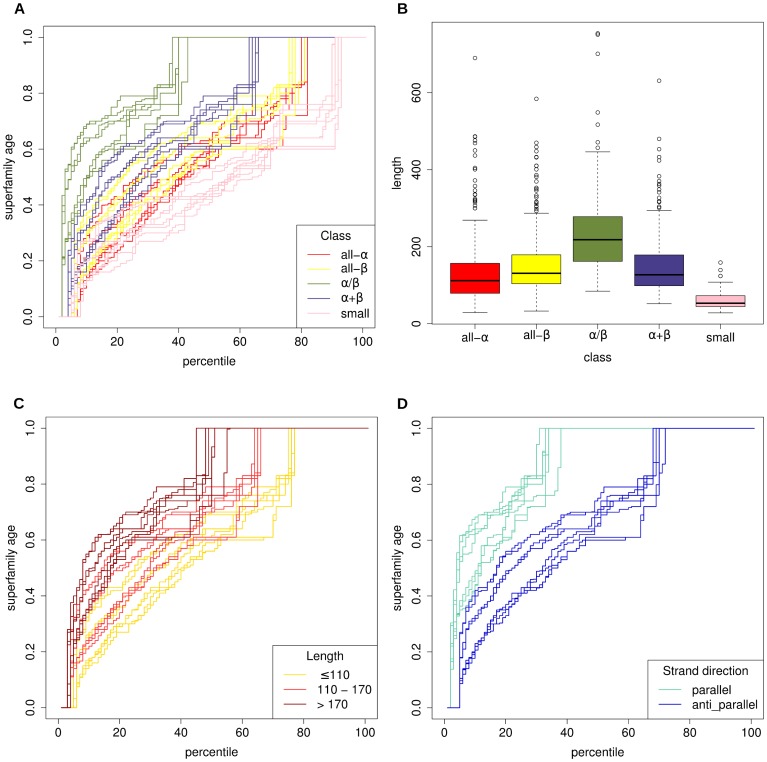
The relationships between superfamily ages, secondary structure and length. Figure A gives a percentile plot of the age distributions of 5 SCOP classes. For ease of interpretation, plots of multi-domain and membrane proteins have been omitted. Each line represents the distribution of ages generated using a different phylogenetic tree. Noticeably, 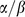 superfamilies' age distributions rise quicker than those of the other classes. Moreover, superfamilies classified as small under SCOP are significantly younger than the other classes. Figure B gives a boxplot of the length distributions for these SCOP classes. Roughly speaking, the ordering of the classes by length corresponds to their ordering by age. 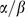 superfamilies are longer and small proteins are shorter than the other classes. Figure C gives a percentile plot of the age distributions of superfamilies with different average domain lengths. Multi-domain superfamilies were omitted from this analysis. Ancient superfamilies are significantly longer than their new-born counterparts. Figure D gives a percentile plot of the age distributions of two populations of superfamilies: those containing a majority parallel strand direction and those with more antiparallel strands. The parallel population is significantly older than the antiparallel superfamilies.


Most globular proteins are classified by their majority secondary structure content in one of the four main SCOP classes (all-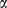, all-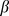, 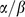 and 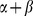). This distinction, while potentially arbitrary from an evolutionary perspective, appears to characterise a large part of the structural variation within fold space [23]. We observe, in consensus with previous work [7], [13], that the age distributions of these classes differ substantially. Figure 2a gives a percentile plot for the age distributions of the SCOP classes. Each line represents the percentiles of an age distributions for a class from a different tree. Most notably, 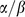 superfamilies appear significantly older than all other SCOP classes (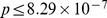). 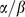 domains tend to be longer than other classes (Figure 2b) and they also contain a large number of the so-called ‘superfolds’: folds containing large numbers of different superfamilies [12].

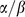
 domains are also unique among the classes in containing a majority of parallel 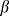-strands as opposed to the antiparallel structure which characterise all-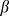 and 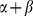 classes. We found that, when looking just at domains with primarily either parallel or antiparallel sheet structure there was a strong, significant preference for superfamilies containing parallel strands to be older than those with antiparallel strands (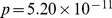, Figure 2c). Parallel sheets are rarely seen containing less than five strands so seem to require the cooperation of a more elaborate hydrogen-bonded network than antiparallel sheets. Parallel strands also tend to have tighter restrictions to the torsion angles of their backbone conformation and tend to be buried by other main chain structures [24].

#### Domain length

Previous studies have demonstrated a significant positive correlation between the length of a domain and its age [7], [14]. The fact that new-born structures appear to be shorter has supported the hypothesis that the primitive protein universe was populated mainly by small folds [7]. We find that ancient superfamilies are significantly longer than new-born superfamilies (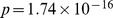, Figure 2d). We also observe that the SCOP class of small proteins significantly younger, than all other classes (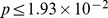, Figure 2a).

The observation that 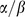 superfamilies are both older and longer than other domains raises the question of whether there are other properties unique to these folds which drive their difference in ages and result in a residual correlation between the length of a domain and its age. In order to investigate this we studied the relationship between domain length and superfamily age stratified by SCOP class.

The relationship between length and age within different classes showed a much weaker correlation than that seen overall. Ancient superfamilies within the all-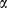 and 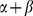 classes still appeared significantly longer than new-born superfamilies within the same classes but other classes failed to show a significant preference (see Figure S3). However, this lack of significance could be due to insufficient numbers of superfamilies in both age groups within these classes. It seems that the relationship between the length of a domain and its age is not purely a residual effect of the age distributions of different SCOP classes.

#### Non-local contacts

We compared the number of non-local contacts with superfamily age and found that ancient superfamilies had significantly more non-local contacts, normalised by radius of gyration, than new-born superfamilies (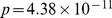). We found no significant difference between the numbers of overall contacts, including local contacts, of ancient and new-born superfamilies. Thus, newly evolved superfamilies appear by this measure to be, on average, simpler and less elaborate structures, with fewer long-range contacts.

#### Buried residues

The residues in the core of a protein structure are key to maintaining the overall architecture of the domain, and its structural stability. There are also more evolutionary constraints on these residues than on surface residues [25].

Here we studied whether there was a correlation between the ages of our superfamilies and the proportion of their residues that were buried. We found that amongst all domains ancient superfamilies contained a significantly higher proportion of buried residues, normalised by the radius of gyration of the structure, than new-born superfamilies (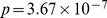). This normalised value for the proportion of buried residues indicates the buried portion of the domain relative to its size. New-born superfamilies therefore tend to have a higher surface area to volume ratio than superfamilies in other age groups.

#### Hydrophobicity

The hydrophobic collapse of a globular polypeptide is thought to be one of the primary forces behind protein folding [2]. The hydrophobicity of the core of a protein structure is thus an important indication of its thermostability and of its folding rate. Given that new-born superfamilies have a higher surface area to volume ratio and there is a marked difference in the hydrophobicities of the core and surface residues in a domain, we investigated whether the age of a domain modulated the hydrophobicity of either its core or its surface.

There was no indication that any age group preferred a highly hydrophilic surface. However, ancient superfamilies tended to contain a more hydrophobic core (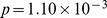) than new-born superfamilies.

#### Disulphide bonds

Another feature that stabilises particular protein structures is the presence of disulphide bonds. These are formed between the thiol groups of two cysteine residues. They are particularly important for the stability of some small proteins and those secreted in the extracellular medium [26]. Here we looked at the age distributions of superfamilies containing disulphide bonds compared to those containing none.

Due to the enrichment of disulphides in extracellular proteins we carried out the analysis using ages estimated by Dollo parsimony from their occurrences in multicellular Eukaryotes only (for details of this see Methods). Even with this constraint superfamilies containing disulphide bonds appear to be significantly younger than those containing none (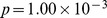). The set of superfamilies containing disulphides contained, as expected, a greater proportion of the small protein class. However, there was no significant difference in the length distributions of superfamilies with disulphide bonds and those containing no disulphide bonds. It is possible that, in new-born superfamilies, disulphide bonds provide extra stability for more simple, less globular structures.

### Sequence level preferences

The enrichment of disulphide bonds among new-born superfamilies indicated a potential over-representation of cysteine residues among these superfamilies. We investigated whether there were further relationships with other amino acids.

Very little is known about the evolution of early life but it is a common theory that the twenty amino acids we see today did not appear simultaneously. It is likely therefore that the earliest peptides consisted of only a subset of these amino acids: the first to evolve. Trifonov suggests a chronological order for the evolution of these amino acids: Gly, Ala, Asp, Val, Pro, Ser, Glu, Leu, Thr, Arg, Ile, Gln, Asn, His, Lys, Cys, Phe, Tyr, Met, Trp [27].

We looked here at the sequence composition of different domains and the propensity for different amino acids for ancient or new-born superfamilies. Since sequence change is rapid compared to structural change it is unlikely that the composition of the earliest peptides could be detected from their extant descendants. However, the propensities calculated here may still hold some signal of preference for certain amino acids.

Propensities were calculated for all 20 amino acids across the two age groups and are shown in Table 1. While amino acids predicted by Trifonov to occur early during protein evolution were more likely to be enriched in ancient superfamilies this relationship was by no means strict. Amino acids significantly over-represented in ancient superfamilies are Arg, Gly, and Val, which are hydrophobic, non-polar residues, with the exception of Arg, which is polar and positively charged. Residues over-represented in new-born superfamilies are Asn, Cys, Gln, Ser, Thr, Trp and Tyr. These residues are mostly polar and uncharged. Trp and Tyr also contain large, aromatic side chains. The propensities in new-born superfamilies for polar residues further supports our previous observation that newly evolving structures may have a larger surface area to volume ratio.

**Table 1:** Preferences of different amino acids for new-born or ancient superfamilies.

amino acid	ancient propensity	p-value	new-born propensity	p-value
Ala	1.03	2.93e-03	**0.94**	4.50e-05
Arg	1.06	2.14e-05	**0.89**	5.59e-09
Asn	**0.91**	2.13e-09	**1.17**	<2.2e-16
Asp	0.97	1.24e-02	1.06	6.03e-04
Cys	**0.84**	4.59e-09	**1.31**	8.88e-16
Gln	**0.92**	3.09e-06	**1.14**	1.57e-10
Glu	1.00	7.46e-01	0.99	6.57e-01
Gly	**1.07**	1.23e-08	**0.88**	5.66e-15
His	1.03	1.18e-01	0.94	3.21e-02
Ile	1.04	2.50e-03	**0.93**	3.37e-05
Leu	1.03	1.34e-02	0.95	6.90e-04
Lys	0.97	1.14e-02	1.06	5.19e-04
Met	1.03	1.95e-01	0.95	7.56e-02
Phe	0.99	4.89e-01	1.02	3.43e-01
Pro	1.03	4.92e-02	0.95	6.97e-03
Ser	**0.93**	1.92e-07	**1.13**	9.15e-13
Thr	0.96	2.50e-03	**1.08**	3.38e-05
Trp	0.91	9.01e-04	**1.18**	5.27e-06
Tyr	0.94	3.69e-04	**1.11**	1.03e-06
Val	**1.05**	2.98e-06	**0.90**	1.46e-10

Propensities for amino acids for a particular age group were calculated using representative domains from the ASTRAL database. P-values were based on a 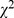-test on the proportions of that amino acid observed in each age group. Values were considered significant and given in bold if the adjusted value (using the Bonferroni correction) was less than 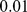. That is, if 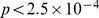.

### Functional preferences

In this study we have primarily focussed on the structural properties characterising superfamilies rather than on their functional roles.

We performed enrichment analysis of GO functions for populations of superfamilies in the different age groups. We compared three different age groups: new-born, ancient and middle-aged superfamilies (those superfamilies in neither the new-born or ancient groups). A list of all terms which were significantly enriched can be found in Table S1.

It has been observed in a study of the protein interaction network of yeast that older proteins tend to have more interaction partners than either middle-aged or young proteins [28]. This would appear to indicate that older superfamilies will tend to have more enriched functional terms than younger superfamilies, since partners in the interaction network will tend to share functional annotations. Indeed we find this to be the case. Of 189 GO terms found to be enriched in any one of the three age groups (ancient, middle-aged or new-born), none were enriched in new-born superfamilies, 8 in middle-aged superfamilies and the remaining 181 were enriched in ancient superfamilies.

The terms enriched in middle-aged superfamilies refer mostly to the regulation of developmental growth unique to Eukaryotes. The majority of terms enriched in ancient superfamilies correspond to fundamental cellular processes common to the vast majority of the tree of life. Interestingly, while RNA synthesis is enriched in ancient superfamilies, terms relating specifically to DNA synthesis are not. This supports the RNA world hypothesis, that during early evolution genetic material was stored as RNA as opposed to DNA [29]. For full details of the functional terms enriched in our age groups see Table S1.

### Does structure or function drive the structural preferences?

We considered the possibility that the structural biases of ancient and new-born superfamilies we report here might be a residual effect of a more fundamental relationship with function. For example, we observe a strong relationship between ancient superfamilies and parallel strands. But, as mentioned before, 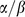 folds are often superfolds, and are known to be associated with a large repertoire of fundamental functions. Perhaps it is the enrichment of these functions in the 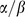 class that drives the preference for ancient superfamilies to have parallel strands.

We compared our structural ages (Figure 2c) with ages for populations of superfamilies annotated with functional terms enriched in either parallel or antiparallel superfamilies. In order to do this we constructed lists of parallel/antiparallel functions: GO terms significantly enriched in the subset of parallel/antiparallel superfamilies. We then compared the ages of the superfamilies annotated with these terms. The results of this comparison are shown in Figure S4. We found that the structural partition resulted in a much more dramatic age difference than the functional groupings. In particular, the functional annotations failed to divide the space efficiently, with many superfamilies annotated with both ‘parallel’ terms and ‘antiparallel’ terms. Even when considering superfamilies unique to a directional functional annotation, there was a less marked distinction than seen in superfamilies distinguished by structural features alone.

### Case study: Common 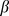-sheet motifs

Not only can these ages be related to general properties of proteins but they also provide a framework for examining more specific questions. For example, we present here a case study for analysing the evolutionary dynamics of certain structural motifs common in domains in a number of different folds.

As was discussed earlier, antiparallel 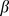-sheet structures appear to be significantly younger than parallel sheets. Antiparallel topologies are, however, more common and more varied than parallel motifs. The most common topology in antiparallel sheets is the hairpin meander where neighbouring strands in a sheet are consecutive in the amino acid sequence. Apart from the simple meander the next two most common topological motifs are the greek key and the jelly roll. Around 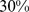 of all-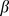 folds in SCOP are annotated as containing either a greek key or a jelly roll and these motifs form a considerable role in their classification. Proteins containing these motifs rarely share either sequence similarity or a common function [30]. The topological architecture of these two common motifs is very similar, with the jelly roll containing a greek key at its core. While some papers treat the jelly roll motif as a special case of the greek key [31], others argue that they occupy a unique portion of fold space [32].

**Figure 3:**
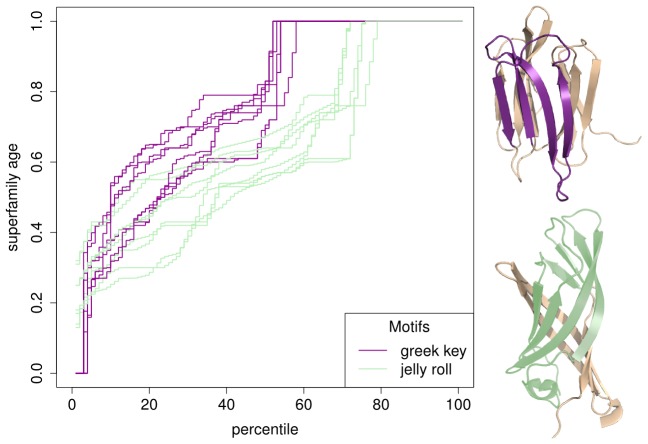
Superfamily ages of greek key and jelly roll motifs. Percentile plots for the age distributions of superfamilies containing a greek key or a jelly roll motif within their beta-sheet topologies. Domains annotated as containing at least one greek key motif are significantly older than those containing the jelly roll motif.

In this study the age distributions of superfamilies classified as containing a greek key or a jelly roll were compared. Greek keys were significantly older than jelly rolls (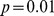, Figure 3). Moreover, we could find no other disparity (for example, in the lengths of these populations) that helped explain this difference.

## Discussion

In this work we estimate the evolutionary age of structural superfamilies. Our results are highly robust to different evolutionary assumptions in estimating ages, as well as alternative topologies and a smaller number of species in the phylogenetic trees.

The results presented here indicate that newly evolving superfamilies tend to be, in general, shorter and structurally more simple than ancient structures. They appear, on average, to have a less hydrophobic core and a greater surface area to volume ratio. They differ from ancient superfamilies in terms of their amino acid composition, containing more polar residues, and tend to contain more additional stabilising features such as disulphide bonds and aromatic residues.

Ancient superfamilies on the other hand are dominated by 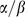 superfamilies and are enriched for many fundamental cellular functions. In particular, the still extant LUCA folds contain a comprehensive repertoire of proteins relating to RNA synthesis and maintenance rather than those used in DNA synthesis, and thus LUCA may have contained a ribosome mechanism for protein synthesis.

The age of a superfamily could also be described as the depth at which it can be traced back through evolution. As such, there are several interpretations of our results, in particular in the case of what we have termed new-born superfamilies. Firstly, it could be that an entirely new domain was formed at some point in evolution. This could indicate that the evolution of a new superfamily as a transition from an already existing structure is a rare event, or that evolutionary transitions through fold space, when they occur, are more often reductive. It could also suggest that, through evolutionary drift, there is a tendency towards an increasingly elaborate structure.

Secondly, a superfamily with a low age estimate might have originated earlier in evolution but the family recognition profiles have failed to identify homologues in distantly related species. In this case, such a superfamily may lack a representative deposition of solved structures, or be rapidly evolving and highly divergent. Certainly, characteristics such as a high solvent accessibility are correlated with the rate of sequence evolution [33]. Nevertheless, by using multiple profiles to build their Hidden Markov Models, SUPERFAMILY improves detection of sequence-divergent families compared to pairwise comparison and single profile searches [34]. As a greater coverage of proteins in such superfamilies are solved structurally, the likelihood of an incorrect low age estimate will decrease.

Thirdly, a young superfamily may be the result of an unfound evolutionary link between superfamilies. As such the structural ancestor of these superfamilies may be earlier than their given age estimates. In order to address this possibility we have shown that the preferences are preserved at both the superfamily and fold level of the SCOP hierarchy.

Finally, what appears to be a young superfamily may actually be ancient but has been lost at several more internal nodes than a parsimonious scenario suggests. This could be the result of functional specialisation within a superfamily. At present our understanding of the evolutionary history of individual superfamilies is not advanced enough to alter the evolutionary model behind age estimation for each superfamily. Our work concerning the robustness of the dataset overall to differing gain weights suggest that our results will be upheld within a moderate level of variation between different superfamilies.

In this study we consider the structural universe of proteins and show that the age preferences of structural characteristics are not a residual effect derived from functional preferences. This result alone justifies the use of protein structures as a fundamental evolutionary unit.

Using our age estimates we examined the specific case of greek key and jelly roll motifs, and identified a significant difference between their ages of origin. Given their similarity in topology it is possible that some superfamilies containing these motifs were involved in evolutionary transitions, where a greek key acted as a scaffold during the innovation of a jelly roll topology.

This example demonstrates that these ages can be used to examine specific properties or motifs of interest, as well as explore more general fold space preferences for proteins at different stages in their evolution.

## Methods

### Superfamily ages

Occurrence profiles of superfamilies across whole genome trees were analysed using the principles of parsimony to estimate when their structural ancestor first evolved. The method described here is based on the the formulation developed by Winstanley et al. [13]. In subsequent sections we outline the process as it is used in this work.

### Superfamily predictions

The data we use in this study were taken primarily from the SUPERFAMILY (v1.75) database. SUPERFAMILY uses families of HMMs to identify homologues of 2,019 SCOP superfamilies. The database comprises protein sequences taken from completely sequenced and annotated genomes and assignments of these sequences to SCOP superfamilies.

We downloaded predicted superfamilies for all 1,496 species available in the SUPERFAMILY database on September 11th 2012. This set was then filtered as follows:

- 407 species annotated as pathogens in the GOLD (v.4) database [35] were removed as pathogens are often associated with incomplete genomes and with lateral gene transfer.
- 31 species which were classified in the category *candidatus*, a provisional status for putative taxa [36] were also removed.
- 44 species found, during the later stages of the method, to lead to poor resolution on the phylogenetic tree were manually identified and removed. These species were largely characterised by having small genomes or were pathogens with annotations missing in the GOLD database and are listed in bold in Table S2.

This left 649 Bacteria, 265 Eukaryotes and 100 Archaea. We called this set the ALLgenomes and it was intended to represent the diversity in the currently known tree of life as accurately as possible. A second set (MULTIgenomes) was created that contained 211 multi-cellular Eukaryotes, a subset of ALLgenomes. The list of all these species including those removed from the original data are included in Table S2.

These predictions of superfamilies on genome sequences were then collapsed to a binary occurrence matrix where each element represents the presence or absence of a superfamily on a genome. A similar occurrence matrix was constructed at the fold level of the SCOP hierarchy.

### Whole genome trees

Multiple species trees were considered as the underlying phylogeny for the completely sequenced genomes. Using numerous trees helps to ensure that the results presented here are robust to inaccuracies in estimating the tree of life. We considered both the NCBI common taxonomy tree [37], [38] as well as phylogenies constructed using the superfamily and fold occurrence profiles calculated above. For completeness the constructed trees were estimated using both parsimony and distance-based algorithms. All the trees were inferred using the PHYLIP package [39]. A total of 8 different trees were constructed for each of the genome sets (ALLgenomes, MULTIgenomes).

#### NCBI trees

The NCBI common taxonomy tree for ALLgenomes and MULTIgenomes were downloaded from the NCBI website. Branch lengths were added using the presence-absence of superfamilies or folds as unweighted, symmetric states using the Wagner parsimony algorithm (PARS) which averages the number of state transitions over all sites and over all possible most parsimonious placements of the state transitions among branches.

#### Distance trees

A neighbour-joining algorithm (NEIGHBOR) was used to construct trees from pairwise distance matrices. The distance metrics used were calculated using a comparison of the numbers of folds or superfamilies on two different genomes. A contingency table was constructed comparing any two genomes 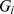 and 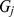. This table counts the number of folds or superfamilies occurring on both genomes (a), those occurring only on 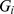 (b), and those occurring just on 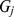 (c).

The distance 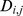 between genomes 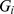 and 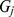 was then calculated using two different dissimilarity metrics defined as follows:

- Jaccard distance: 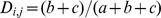

- Bray-Curtis distance: 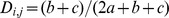

Matrices were composed of the distances between every pairwise combination of species in a set and used as input to the tree building algorithm. For each genome set four distance matrices were calculated: using the Jaccard and the Bray-Curtis distances on superfamily and fold occurrence data.

In all these cases, an extended majority rule consensus tree (CONSENSE) was calculated from individual trees constructed using neighbour-joining on 100 delete-half jackknife samples of the original occurrence data. Branch lengths were added to this consensus topology using the Fitch-Margoliash algorithm (FITCH) using the complete distance matrix.

#### Parsimony trees

Trees were also built using Wagner parsimony (PARS) and treating the presence-absence data of folds or superfamilies as unweighted, symmetric states. Extended majority-rule consensus trees (CONSENSE) were summarised from trees built from 100 delete-half jackknife samples of the occurrence data where up to 10 trees tied for the best parsimony score were retained per sample. Branch lengths were added to the consensus trees using a final implementation of the Wagner parsimony algorithm (PARS).

#### Tree transformations

The trees for ALLgenomes were rooted at the trifurcation of the three superkingdoms and the trees for MULTIgenomes were rooted by including the archaeal species *Acidianus hospitalis* and using this as an outgroup. Branch lengths were normalised to lie between 0 and 1, with the root at 0 and the leaves at 1.

### Age estimation

For each tree, the age of a superfamily is the result of a parsimony analysis on potential gain and loss events of the superfamily.

#### Maximum parsimony

The maximum parsimony analysis was undertaken as implemented by Mirkin et al. [16]. Given the occurrence profile of a superfamily across the genomes, several scenarios of gain and loss events at internal and external nodes of the tree can be proposed which explain the profile. Maximum parsimony attempts to find the scenario which minimises the score 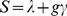, where 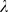 and 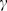 are the numbers of loss and gain events respectively and 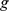 is the gain weight.

By minimising this score the algorithm considers vertical descent of superfamilies to be by far the most common evolutionary scenario at any species-ization event on the tree. Both lateral gene transfer and de novo gene gain are considered as gain events and the likelihood of these events occurring, relative to gene loss, is parametrised as the gain weight 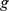. For this work we primarily used a gain weight of 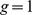, maintaining an equal penalty for both loss and gain events. Further analysis was also carried out using values of 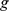 ranging from 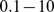 incorporating up to a 10-fold penalty on either loss events or gain events relative to each other.

#### Dollo parsimony

On the trees of MULTIgenomes species Dollo parsimony was adopted as the default model for age estimation. Dollo parsimony allows at most a single gain event and aims to minimise the number of subsequent loss events.

#### Fusion parsimony

The maximum parsimony model described above was adjusted to allow at most one gain event to occur on the Eukaryotic subtree. As such, fusion parsimony assumes Dollo parsimony on Eukaryotic genomes and maximum parsimony elsewhere as the most likely evolutionary model for domain evolution.

Relative ages are quantified as the height of the node of the earliest event and as such are a number between 0 and 1, where an age of 0 refers to a superfamily whose structural ancestor first appeared on one or more leaves of the tree and an age of 1 refers to a superfamily whose structural ancestor first appeared before the trifurcation of the superkingdoms.

#### Age groups

There are 557 ancient superfamilies, assigned a relative age of 1, and 443 superfamilies with an age 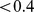, which are referred to as new-born superfamilies. The value for this cut-off was primarily chosen to allow for comparable numbers of superfamilies in the new-born and ancient subsets. Where applicable, middle-aged superfamilies are any superfamily not counted as ancient or new-born. The distribution of superfamily ages is given in Figure S5.

### Fold space preferences

Structural properties of 1,279 superfamilies were obtained using domains from the ASTRAL (1.75) database [22] with an aerospaci score 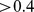 and filtered to 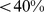 sequence identity. This set of 5,493 domains will be referred to as the ASTRAL40 set. The number of representative ASTRAL40 domains for each superfamily is included in Table S3.

Comparisons between the properties of new-born and ancient superfamilies were carried out using the Mann-Whitney U test [40]. Since multiple superfamilies shared the same age and therefore tied in rank the standard deviation of the distribution for the test statistic was appropriately adjusted [41].

While age distributions from all trees were considered in the analysis, for simplicity the p-values reported in the Results section derive from the ages calculated by maximum parsimony on the NCBI tree with branch lengths added using superfamily annotations. However, the results are only reported as significant if they gave significant p-values on ages from all the trees.

#### Length

Lengths of superfamilies were defined as the mean of the sequence length of domains representing that superfamily in the ASTRAL40 set. Superfamilies classified as multi-domain proteins in SCOP were omitted from this analysis.

#### Strand direction

Secondary structure was assigned using DSSP [42] and the direction of a strand relative to each of its hydrogen bonding partners was calculated using PROMOTIF [43]. Only domains in the ASTRAL40 set with 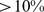 strand content were considered. Each domain was then annotated as parallel if 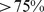 of it's strand residues were in parallel strands, antiparallel if 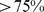 of its strand residues were in antiparallel strands and mixed otherwise. The label for a superfamily was summarised as the majority label for its representative domains.

#### Non-local contacts

Two residues were said to be in contact if their 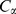 atoms are 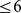 Å apart (see,for example [44]). Contacts are defined as non-local if they occur between atoms 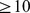 residues apart. The number of non-local contacts for a domain is normalised by dividing by its radius of gyration:
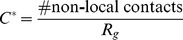
Non-local contacts were summarised for a superfamily as the mean value of 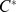 on its representative domains.

#### Radius of gyration

The centre of mass (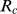) and the radius of gyration (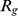) of a domain were calculated from the coordinates of the 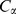 atoms (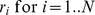):
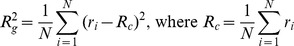



#### Buried residues

The solvent accessibility of a residue was assigned using JOY [45]. A residue was classified as buried if 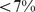 of its surface area is exposed to water. The proportion of buried residues in a domain of length 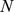 was normalised by the radius of gyration, an estimate of the volume of the structure:
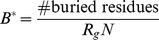
Buried residues for a superfamily were generalised as the mean value of 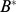 across its representative domains.

#### Hydrophobicity

The hydrophobicity of a residue was measured using the OMH scale [46]. The hydrophobicity of a sequence of amino acids was calculated as the sum of hydrophobicities of each residue divided by the length of the sequence. Summary values for the hydrophobicity of a superfamily were calculated by averaging over the hydrophobicities of its representative domains in the ASTRAL40 set.

#### Disulphide bonds

Disulphide bonds were annotated with JOY [45]. Each domain in the ASTRAL40 set was annotated as to whether it contained disulphide bonds or not. If more than half of the representative domains for a particular superfamily contained at least one disulphide bond it was counted as a superfamily with disulphide bonds. A superfamily was considered to contain no disulphide bonds only if all its domains in the ASTRAL40 set contained no disulphide bonds.

#### Amino acid content

The Propensities of an amino acid 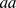 for ancient and new-born domains were calculated as:
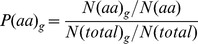
where 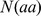 is the number of amino acids of type 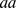 across all domains in the ASTRAL40 set, 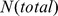 is the total number of amino acids in these domains, and 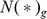 is the number of amino acids in domains representing superfamilies predicted to belong to an age group 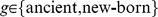. Propensities have an expected value of 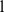, with values 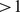 indicating over-representation of that amino acid in a particular age group compared to the background distribution and values 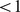 indicating under-representation. We calculated the significance of these propensities using a 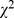-test with a single degree of freedom on the observed occurrences of that amino acid in that age group 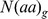. To account for multiple testing the Bonferroni correction was used and only propensities with 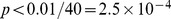 were considered significant.

#### Function

GO functional annotations [47] for SCOP superfamilies were downloaded from the SUPERFAMILY website [34]. These functional annotations were assembled using GO terms assigned to Uniprot proteins [48] with known SCOP classifications.

Functional enrichment analysis was performed on this set, assuming the number of superfamilies annotated with a particular GO term followed a hypergeometric distribution [49], and significance calculated with a one-sided test for the enrichment of a term in a particular age group 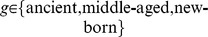. As above, the Bonferroni correction was used to account for multiple testing. A total of 7,394 GO terms were investigated so terms with a 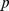-value 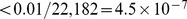 were considered significant.

#### Greek key and jelly roll motifs

Greek key motifs were extracted from ASTRAL40 domains using the method outlined in [30]. Strand hydrogen bond partners were assigned using PROMOTIF [43]. As the jelly roll motif is formed by adding two extra strands to a greek key motif, these were then identified from the greek key set. Superfamilies with a jelly roll motif found in any representative domain contributed to the jelly roll set. All other superfamilies containing domains annotated with a greek key motif were counted as the greek key set. The result was 105 superfamilies containing a greek key motif and 33 containing a jelly roll.

## Supporting Information

Figure S1 **The effect of altering the gain weight on fold space preferences.** Fold space preferences were recalculated using ages generated on the NCBI tree using a maximum parsimony algorithm with different gain weights. The gain weight represents the relative penalty of gain events as opposed to loss events in a superfamily's evolutionary history. By altering the gain weight between 0.1 and 10 we explore up to a 10-fold asymmetry in the likelihood of these two events. The quantile plots here show the results of an analysis of SCOP class, strand direction and domain length against ages generated with these different gain weights.
(TIF)

Figure S2 **The effect of altering the parsimony model on fold space preferences.** Fold space preferences were recalculated using ages generated using a fusion parsimony algorithm on the NCBI tree. This fusion model assigned gain and loss events at internal nodes of the tree according to maximum parsimony on the Bacterial and Archaeal subtrees and according to Dollo parsimony on the Eukaryotic subtree. The quantile plots here show the results of an analysis of SCOP class, strand direction and domain length against ages generated using either a maximum or a fusion parsimony analysis.
(TIF)

Figure S3
**Domain lengths and their relationship to superfamily age when stratified by their class.** Percentile plots of the ages for different domain lengths within the four main SCOP classes. Ancient domains are significantly longer than new-born domains in both the all-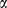 and the 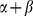 classes but not in the all-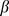 and 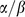 classes. The ages shown are calculated using a maximum parsimony algorithm on the NCBI tree.(TIF)

Figure S4 **Structure vs. functional annotations on fold space preferences.** Three percentile plots exploring the differences between superfamilies with parallel or antiparallel beta-sheet structure. The structural annotation plot shows the age distributions of superfamilies with a majority of either parallel or antiparallel strands. It is a reproduction of Figure 2D. The functional annotation plots compare the age distributions of superfamilies annotated with *parallel* or *antiparallel* functions: that is functional terms significantly enriched in the parallel or antiparallel set of superfamilies. The functional annotations fail to divide the space effectively with 758 superfamilies annoted with both *parallel* and *antiparallel* functions. When considering superfamilies unique to a directional functional annotation there appeared a less marked distinction in the age distributions than was shown using the structural annotation.
(TIF)

Figure S5
**The distribution of ages.** Histograms are drawn for the distribution of superfamily ages across all 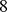 trees built using occurrences on the 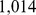 ALLgenomes. Tree names reference the method used to construct the topology (NCBI common taxonomy tree (NCBI), Neighbour-joining with Jaccard distances (JACC), Neighbour-joining with Bray-Curtis distances (BC), and Wagner Parsimony (PARS)) and whether it was constructed using superfamily or fold (F) occurrences on the genomes. Ages were calculated using either a maximum parsimony algorithm with the probability of a gain and loss event equally weighted, or a fusion parsimony algorithm (see methods).(TIF)

Table S1 **Enriched functional terms for different age groups.** GO terms that are found to be significantly enriched in new-born, middle-aged, or ancient superfamilies. Terms in italics are supported by analysis on annotations derived purely from single domain Uniprots only. These terms can be understood as domain-centric functional annotations but as they are more rare they lead to a less specific enrichment analysis.
(PDF)

Table S2 **List of complete genomes.** The list of species names used for superfamily predictions and tree building. Species in italics were removed from the data set as pathogens or Candidatus species. Species in bold were removed manually.
(PDF)

Table S3 **ASTRAL40 domains.** The number of domains for each superfamily with representative structures in the ASTRAL40 set.
(PDF)
