# Supplementary figures and images for: Exploring Fold Space Preferences of New-born and Ancient Protein Superfamilies
Source: PLoS Comput Biol. 2013 Nov 14;9(11):e1003325. doi: 10.1371/journal.pcbi.1003325 (PMC3828129; doi:10.1371/journal.pcbi.1003325)

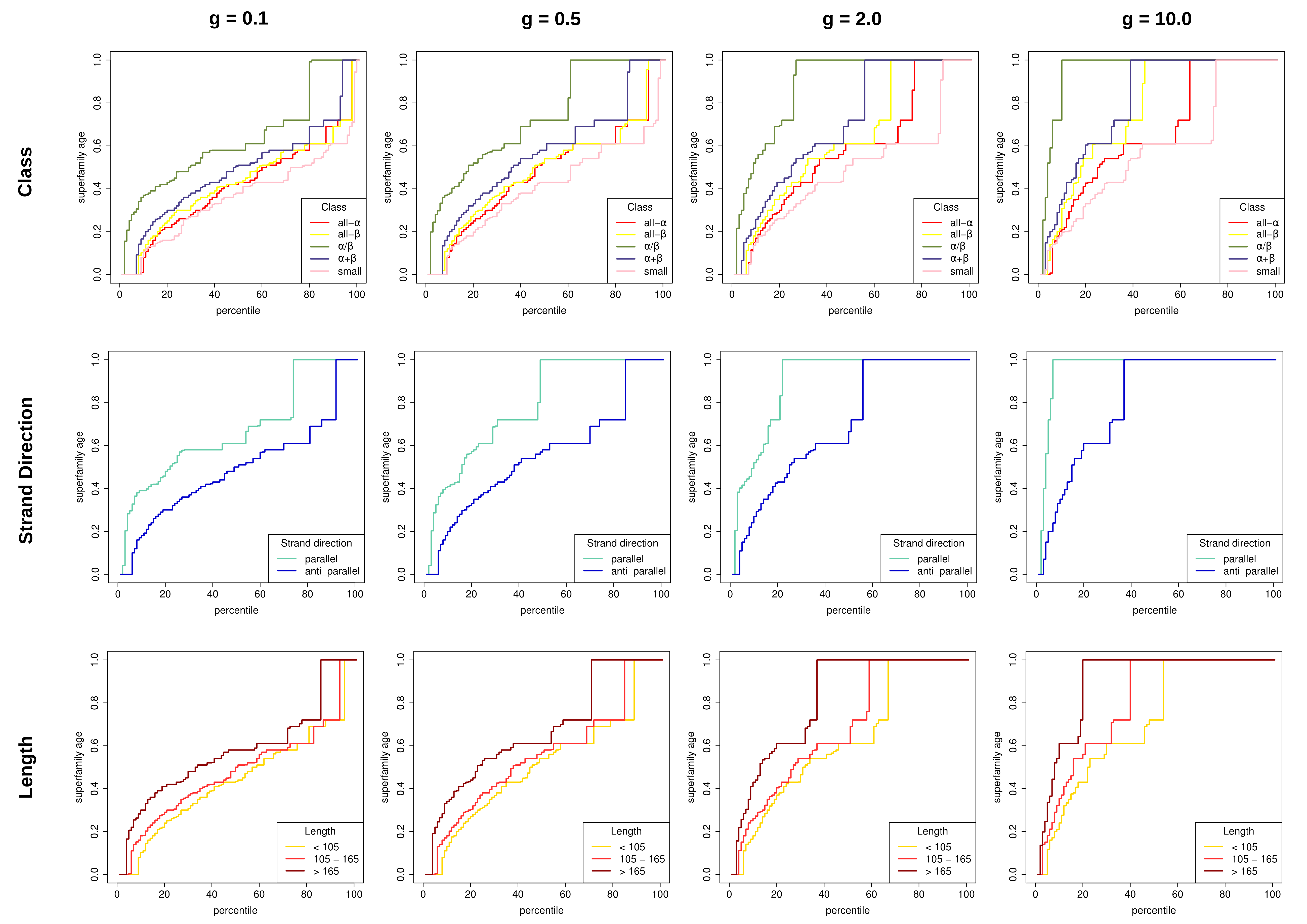

Supplement: Figure S1 — The effect of altering the gain weight on fold space preferences. Fold space preferences were recalculated using ages generated on the NCBI tree using a maximum parsimony algorithm with different gain weights. The gain weight represents the relative penalty of gain events as opposed to loss events in a superfamily's evolutionary history. By altering the gain weight between 0.1 and 10 we explore up to a 10-fold asymmetry in the likelihood of these two events. The quantile plots here show the results of an analysis of SCOP class, strand direction and domain length against ages generated with these different gain weights. (TIF) [file pcbi.1003325.s001.tif]

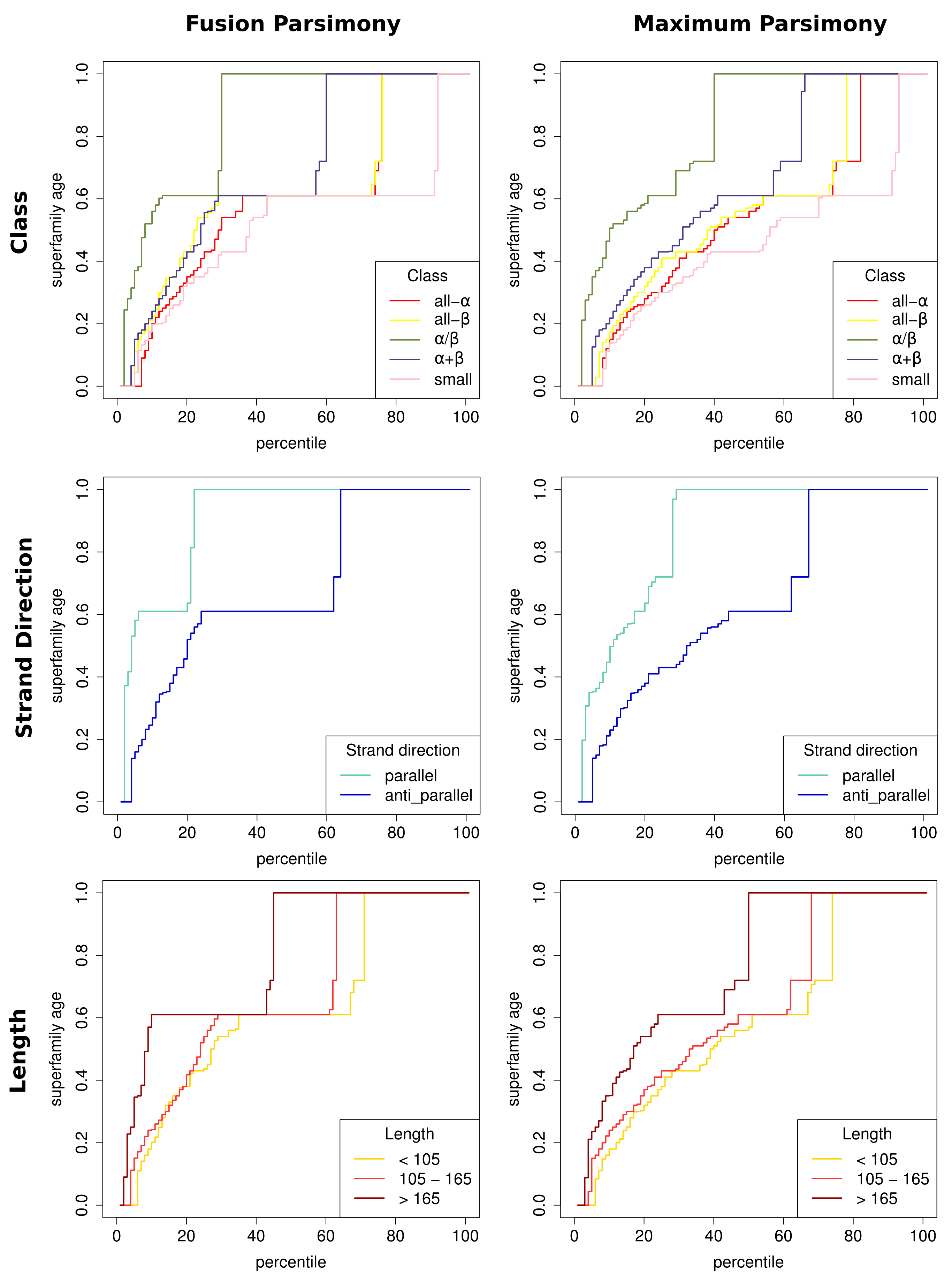

Supplement: Figure S2 — The effect of altering the parsimony model on fold space preferences. Fold space preferences were recalculated using ages generated using a fusion parsimony algorithm on the NCBI tree. This fusion model assigned gain and loss events at internal nodes of the tree according to maximum parsimony on the Bacterial and Archaeal subtrees and according to Dollo parsimony on the Eukaryotic subtree. The quantile plots here show the results of an analysis of SCOP class, strand direction and domain length against ages generated using either a maximum or a fusion parsimony analysis. (TIF) [file pcbi.1003325.s002.tif]

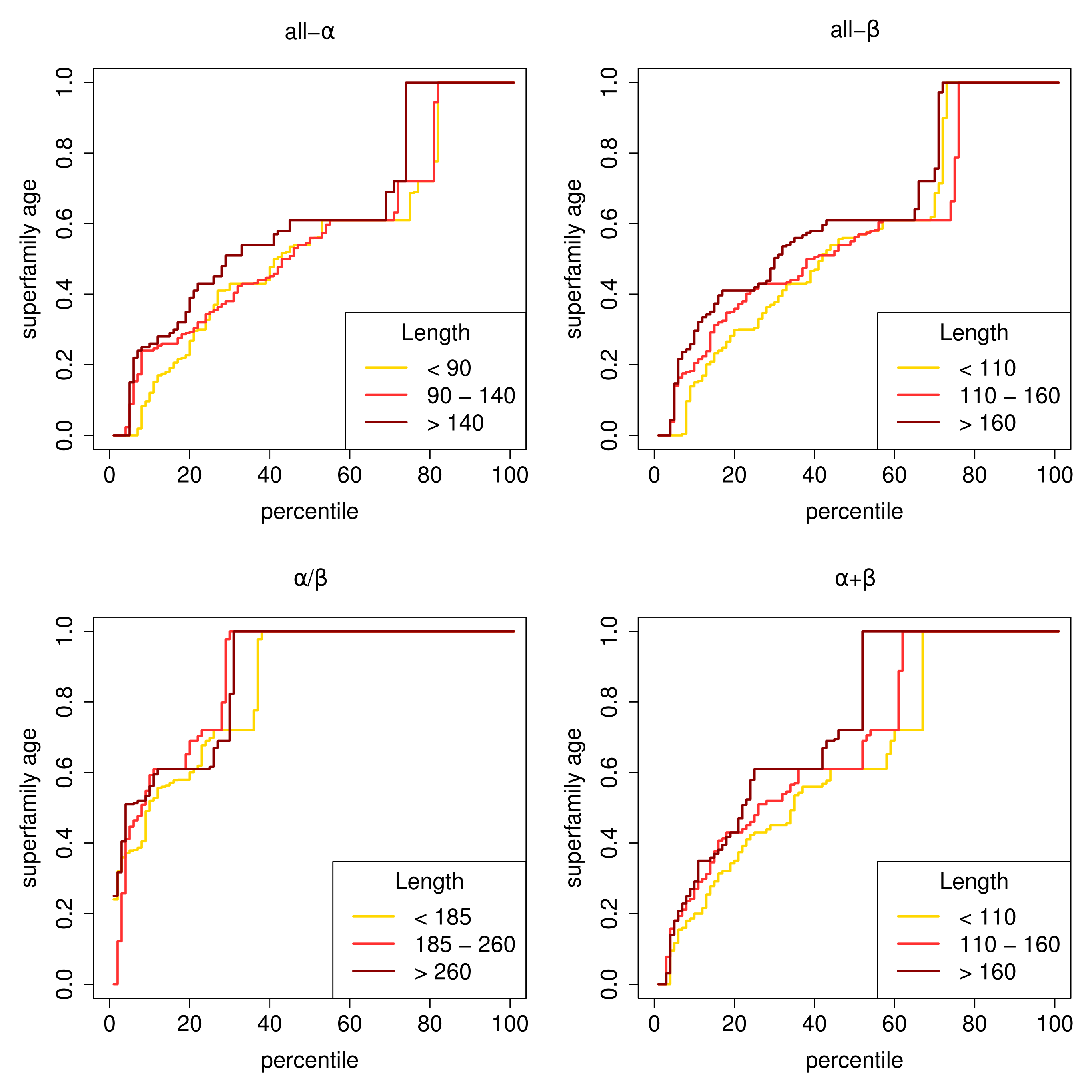

Supplement: Figure S3 — Domain lengths and their relationship to superfamily age when stratified by their class. Percentile plots of the ages for different domain lengths within the four main SCOP classes. Ancient domains are significantly longer than new-born domains in both the all- and the classes but not in the all- and classes. The ages shown are calculated using a maximum parsimony algorithm on the NCBI tree. (TIF) [file pcbi.1003325.s003.tif]

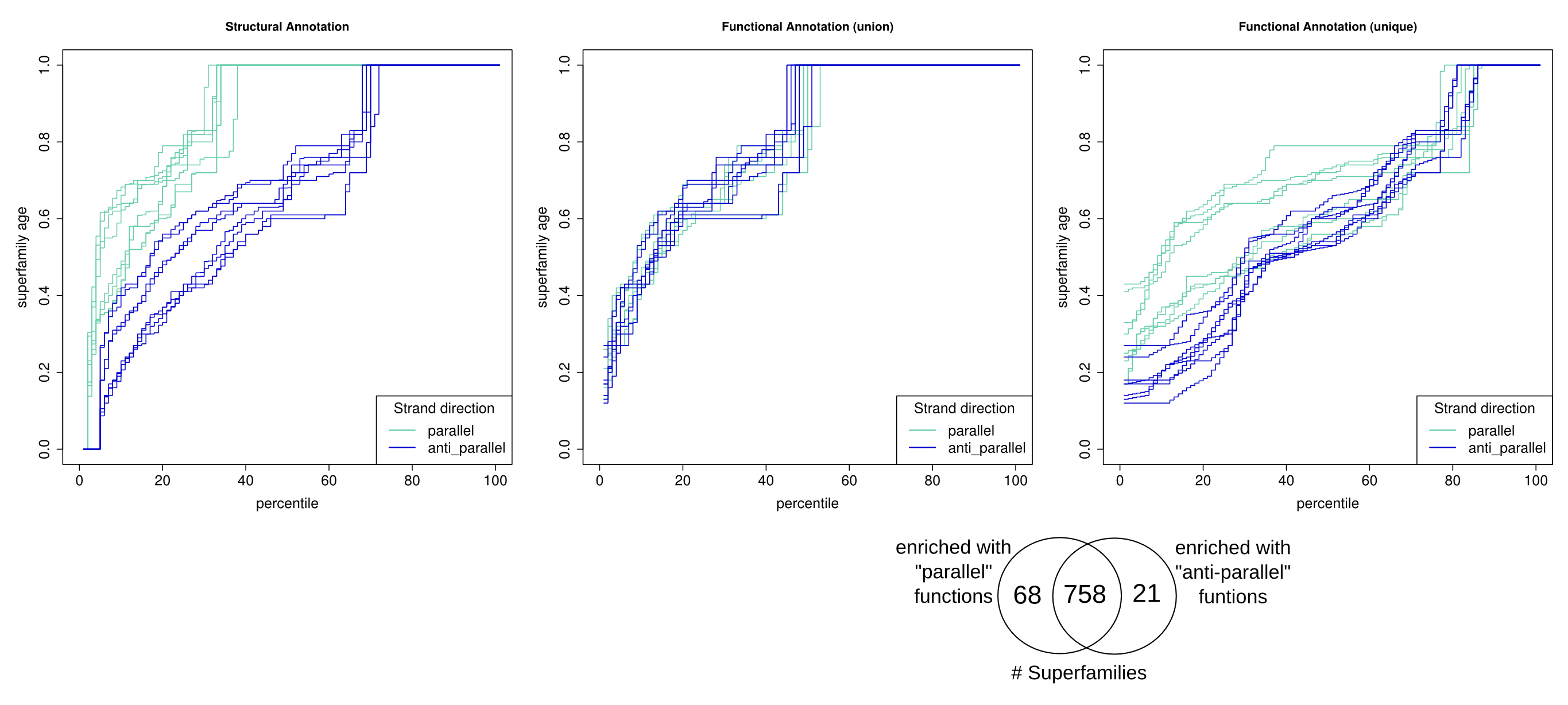

Supplement: Figure S4 — Structure vs. functional annotations on fold space preferences. Three percentile plots exploring the differences between superfamilies with parallel or antiparallel beta-sheet structure. The structural annotation plot shows the age distributions of superfamilies with a majority of either parallel or antiparallel strands. It is a reproduction of Figure 2D. The functional annotation plots compare the age distributions of superfamilies annotated with parallel or antiparallel functions: that is functional terms significantly enriched in the parallel or antiparallel set of superfamilies. The functional annotations fail to divide the space effectively with 758 superfamilies annoted with both parallel and antiparallel functions. When considering superfamilies unique to a directional functional annotation there appeared a less marked distinction in the age distributions than was shown using the structural annotation. (TIF) [file pcbi.1003325.s004.tif]

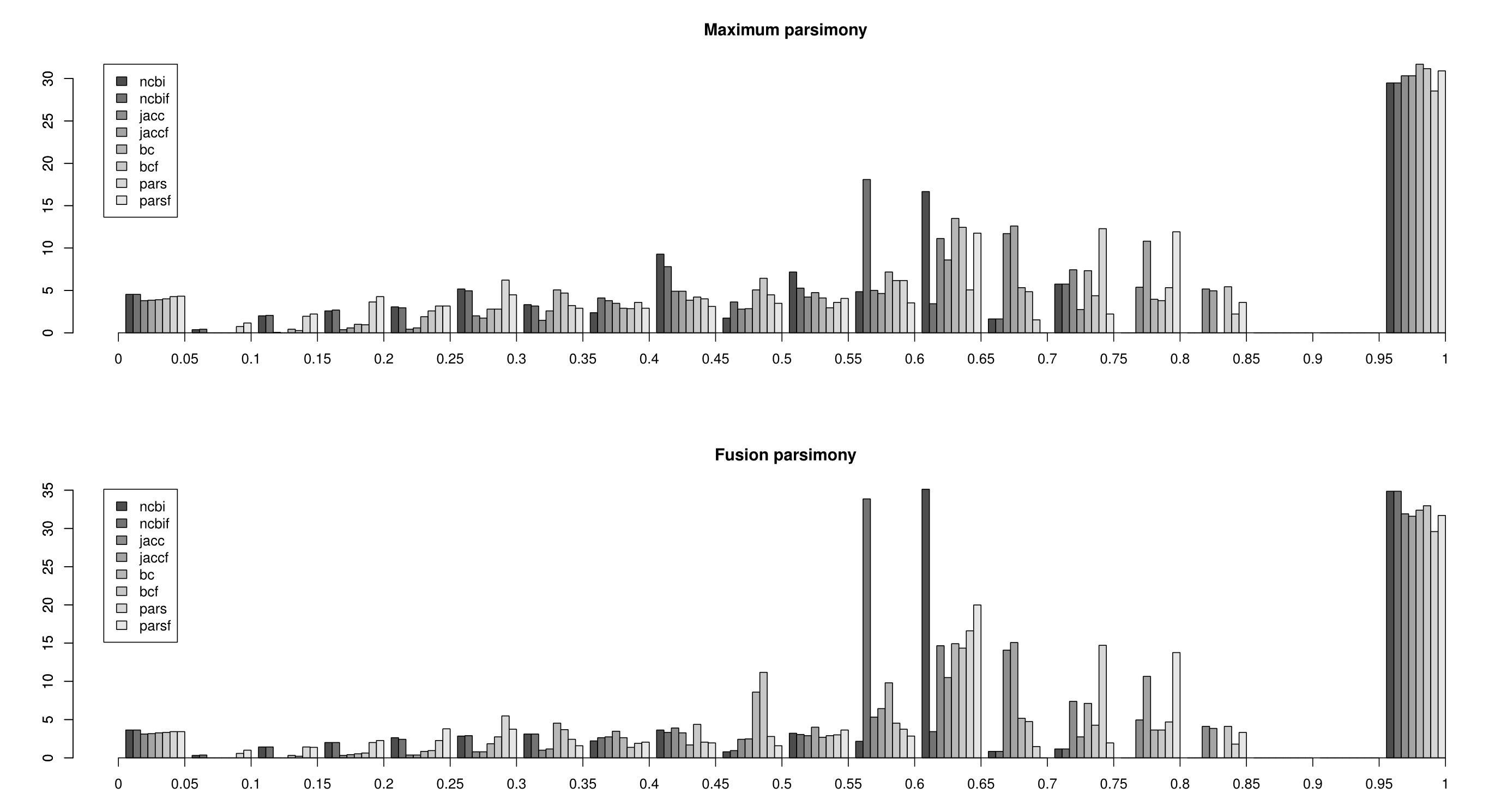

Supplement: Figure S5 — The distribution of ages. Histograms are drawn for the distribution of superfamily ages across all trees built using occurrences on the ALLgenomes. Tree names reference the method used to construct the topology (NCBI common taxonomy tree (NCBI), Neighbour-joining with Jaccard distances (JACC), Neighbour-joining with Bray-Curtis distances (BC), and Wagner Parsimony (PARS)) and whether it was constructed using superfamily or fold (F) occurrences on the genomes. Ages were calculated using either a maximum parsimony algorithm with the probability of a gain and loss event equally weighted, or a fusion parsimony algorithm (see methods). (TIF) [file pcbi.1003325.s005.tif]
