# Supplementary material for: Exploring Fold Space Preferences of New-born and Ancient Protein Superfamilies
Source: PLoS Comput Biol. 2013 Nov 14;9(11):e1003325. doi: 10.1371/journal.pcbi.1003325 (PMC3828129; doi:10.1371/journal.pcbi.1003325)
Supplement: Table S1 — Enriched functional terms for different age groups. GO terms that are found to be significantly enriched in new-born, middle-aged, or ancient superfamilies. Terms in italics are supported by analysis on annotations derived purely from single domain Uniprots only. These terms can be understood as domain-centric functional annotations but as they are more rare they lead to a less specific enrichment analysis. (PDF) [file pcbi.1003325.s006.pdf]

Table S1. Enriched functional terms for different age groups.

GO term	Age group	p-value
anatomical structure morphogenesis	middle_aged	0.00727
positive regulation of cellular process	middle_aged	0.00346
protein binding	middle_aged	0.00631
regulation of cellular process	middle_aged	0.0095
regulation of developmental process	middle_aged	0.00103
tissue development	middle_aged	0.00645
acetyl-CoA metabolic process	ancient	8.35e-05
adenyl nucleotide binding	ancient	0.00169
adenyl ribonucleotide binding	ancient	0.00169
amine biosynthetic process	ancient	3.26e-09
amine catabolic process	ancient	0.00225
amine metabolic process	ancient	5.41e-21
aspartate family amino acid metabolic process	ancient	0.00193
ATP metabolic process	ancient	0.00744
biosynthetic process	ancient	2.36e-34
carbohydrate biosynthetic process	ancient	1.45e-08
carbohydrate catabolic process	ancient	0.00017
carbohydrate derivative catabolic process	ancient	0.000489
carbohydrate derivative metabolic process	ancient	8.94e-09
carbohydrate metabolic process	ancient	1.4e-05
carbon-carbon lyase activity	ancient	3.54e-06
carbon-oxygen lyase activity	ancient	0.00221
carboxylic acid biosynthetic process	ancient	4.16e-08
carboxylic acid metabolic process	ancient	1.03e-21
carboxy-lyase activity	ancient	0.00424
catabolic process	ancient	2.38e-13
catalytic activity	ancient	7.25e-35
cellular amine metabolic process	ancient	3.17e-23
cellular amino acid biosynthetic process	ancient	1.26e-09
cellular amino acid metabolic process	ancient	1.16e-20
cellular aromatic compound metabolic process	ancient	1.97e-10
cellular biosynthetic process	ancient	1.27e-30
cellular carbohydrate biosynthetic process	ancient	0.00193
cellular catabolic process	ancient	4.69e-09
cellular component biogenesis at cellular level	ancient	0.000489
cellular ketone metabolic process	ancient	2.96e-23
cellular macromolecule biosynthetic process	ancient	8.81e-09
cellular macromolecule metabolic process	ancient	5.46e-06
cellular metabolic process	ancient	7.08e-39
cellular nitrogen compound biosynthetic process	ancient	5.57e-21
cellular nitrogen compound catabolic process	ancient	3.01e-05
cellular nitrogen compound metabolic process	ancient	1.43e-28
cellular polysaccharide biosynthetic process	ancient	0.00424
cellular polysaccharide metabolic process	ancient	0.000883
cellular process	ancient	1.56e-14

Continued on next page

Table S1 – continued from previous page

GO term	Age group	p-value
cellular respiration	ancient	0.0081
coenzyme biosynthetic process	ancient	2.77e-08
coenzyme metabolic process	ancient	7.84e-20
cofactor biosynthetic process	ancient	3.26e-08
cofactor catabolic process	ancient	0.00193
cofactor metabolic process	ancient	1.9e-17
cytoplasm	ancient	8.9e-09
cytoplasmic part	ancient	1.52e-10
cytosol	ancient	0.000178
cytosolic part	ancient	1.63e-06
dicarboxylic acid metabolic process	ancient	0.000403
energy derivation by oxidation of organic compounds	ancient	6.56e-06
gene expression	ancient	2.2e-08
generation of precursor metabolites and energy	ancient	6.03e-10
glucose catabolic process	ancient	0.000183
glucose metabolic process	ancient	3.8e-05
glutamine family amino acid metabolic process	ancient	0.000382
heterocycle biosynthetic process	ancient	9.02e-15
heterocycle catabolic process	ancient	6.34e-05
heterocycle metabolic process	ancient	5.49e-28
hexose catabolic process	ancient	1.72e-05
hexose metabolic process	ancient	2.9e-07
hydrolase activity	ancient	8.29e-07
hydrolase activity, acting on acid anhydrides	ancient	0.000662
hydrolase activity, acting on acid anhydrides, in phosphorus-containing anhydrides	ancient	0.000662
hydrolase activity, acting on carbon-nitrogen (but not peptide) bonds	ancient	0.0081
intracellular	ancient	0.00112
intracellular part	ancient	0.00112
isomerase activity	ancient	0.00308
large ribosomal subunit	ancient	0.00108
lyase activity	ancient	7.91e-10
macromolecule biosynthetic process	ancient	9.97e-09
macromolecule metabolic process	ancient	0.00217
metabolic process	ancient	2.95e-35
microbody	ancient	0.00928
mitochondrial matrix	ancient	1.02e-07
mitochondrial part	ancient	2.73e-08
mitochondrion	ancient	1.23e-14
molecular function	ancient	7.68e-08
monocarboxylic acid metabolic process	ancient	0.00421
monosaccharide catabolic process	ancient	3.54e-06
monosaccharide metabolic process	ancient	2.11e-07
ncRNA metabolic process	ancient	8.14e-07
ncRNA processing	ancient	8.43e-05
nicotinamide nucleotide metabolic process	ancient	0.00926

Continued on next page

Table S1 – continued from previous page

GO term	Age group	p-value
nitrogen compound metabolic process	ancient	1.37e-26
nucleobase-containing compound biosynthetic process	ancient	1.32e-08
nucleobase-containing compound catabolic process	ancient	7.16e-05
nucleobase-containing compound metabolic process	ancient	1.56e-13
nucleobase-containing small molecule metabolic process	ancient	3.05e-23
nucleoside metabolic process	ancient	0.000124
nucleoside phosphate metabolic process	ancient	1.56e-20
nucleoside triphosphate catabolic process	ancient	0.00216
nucleoside triphosphate metabolic process	ancient	2.37e-05
nucleotide binding	ancient	3.1e-06
nucleotide biosynthetic process	ancient	4.32e-05
nucleotide catabolic process	ancient	0.000317
nucleotide metabolic process	ancient	3.32e-20
organic acid biosynthetic process	ancient	4.16e-08
organic acid catabolic process	ancient	0.00639
organic acid metabolic process	ancient	1.23e-22
organic substance metabolic process	ancient	3.19e-08
oxidation-reduction process	ancient	1.16e-08
oxidoreductase activity	ancient	1.46e-06
oxidoreductase activity, acting on CH-OH group of donors	ancient	0.000128
oxidoreductase activity, acting on the CH-OH group of donors, NAD or NADP as acceptor	ancient	0.00193
oxidoreduction coenzyme metabolic process	ancient	0.000403
oxoacid metabolic process	ancient	2.51e-22
plastid	ancient	0.000132
polysaccharide biosynthetic process	ancient	8.35e-05
primary metabolic process	ancient	3.35e-24
purine-containing compound biosynthetic process	ancient	6.18e-05
purine-containing compound metabolic process	ancient	6.56e-13
purine nucleoside metabolic process	ancient	0.00313
purine nucleoside triphosphate catabolic process	ancient	0.00803
purine nucleoside triphosphate metabolic process	ancient	0.000325
purine nucleotide binding	ancient	0.000327
purine nucleotide biosynthetic process	ancient	0.0038
purine nucleotide catabolic process	ancient	0.00111
purine nucleotide metabolic process	ancient	1.6e-11
purine ribonucleoside metabolic process	ancient	0.00313
purine ribonucleoside triphosphate metabolic process	ancient	0.000619
purine ribonucleotide binding	ancient	0.000327
purine ribonucleotide catabolic process	ancient	0.00417
purine ribonucleotide metabolic process	ancient	3.26e-09
pyridine-containing compound metabolic process	ancient	0.00424
pyridine nucleotide metabolic process	ancient	0.00424
pyrophosphatase activity	ancient	0.00111
ribonucleoprotein complex	ancient	0.00565
ribonucleoside metabolic process	ancient	0.000382

Continued on next page

Table S1 – continued from previous page

**GO term**	**Age group**	**p-value**
ribonucleoside triphosphate metabolic process	ancient	0.000325
ribonucleotide binding	ancient	0.000327
ribonucleotide catabolic process	ancient	0.00417
ribonucleotide metabolic process	ancient	4.23e-09
ribosomal subunit	ancient	1.57e-09
ribosome	ancient	5.52e-09
RNA binding	ancient	0.00321
small molecule binding	ancient	2.74e-05
small molecule biosynthetic process	ancient	1.15e-09
small molecule catabolic process	ancient	0.0019
small molecule metabolic process	ancient	3.4e-34
structural constituent of ribosome	ancient	3.01e-08
sulfur compound biosynthetic process	ancient	0.00221
sulfur compound metabolic process	ancient	4.51e-05
transferase activity	ancient	2.35e-07
translation	ancient	8.11e-10
tRNA metabolic process	ancient	3.19e-07
