# Supplementary material for: Exploring Fold Space Preferences of New-born and Ancient Protein Superfamilies
Source: PLoS Comput Biol. 2013 Nov 14;9(11):e1003325. doi: 10.1371/journal.pcbi.1003325 (PMC3828129; doi:10.1371/journal.pcbi.1003325)
Supplement: Table S2 — List of complete genomes. The list of species names used for superfamily predictions and tree building. Species in italics were removed from the data set as pathogens or Candidatus species. Species in bold were removed manually. (PDF) [file pcbi.1003325.s007.pdf]

Table S2. List of complete genomes.

**Taxa**	**Abbreviation**
Acaryochloris marina MBIC11017	Removed: Pathogen
Acetobacter pasteurianus IFO 3283-01	BPrAl_Apas
Acetobacterium woodii DSM 1030	BFiCl_Awoo
Acetohalobium arabaticum DSM 5501	BFiCl_Aara
**Acholeplasma laidlawii PG-8A**	Removed
Achromobacter xylosoxidans A8	BPrBe_Axyl
Acidaminococcus fermentans DSM 20731	BFiNe_Afer
Acidaminococcus intestini RyC-MR95	BFiNe_Aint
Acidianus hospitalis W1	ACrTh_Ahos
Acidilobus saccharovorans 345-15	ACrTh_Asac
Acidimicrobium ferrooxidans DSM 10331	BAcAc_Afer
Acidiphilium cryptum JF-5	Removed: Pathogen
Acidiphilium multivorum AIU301	BPrAl_Amul
Acidithiobacillus caldus SM-1	BPrGa_Acal
Acidithiobacillus ferrivorans SS3	BPrGa_Afer
Acidithiobacillus ferrooxidans ATCC 53993	BPrGa_Afeo
Acidobacterium capsulatum ATCC 51196	BFiAc_Acap
Acidothermus cellulolyticus 11B	Removed: Pathogen
Acidovorax avenae subsp. avenae ATCC 19860	Removed: Pathogen
Acidovorax citrulli AAC00-1	Removed: Pathogen
Acidovorax ebreus TPSY	BPrBe_Aebr
Acidovorax sp. JS42	Removed: Pathogen
Aciduliprofundum boonei T469	AEuun_Aboo
Acinetobacter baumannii ATCC 17978	Removed: Pathogen
Acinetobacter calcoaceticus PHEA-2	Removed: Pathogen
Acinetobacter oleivorans DR1	Removed: Pathogen
Acinetobacter sp. ADP1	Removed: Pathogen
Acremonium alcalophilum	EFuDi_Aalc
Acromyrmex echinatior	EOpMe_Aech
Acropora digitifera	EMeCn_Adig
Actinobacillus pleuropneumoniae serovar 5b str. L20	Removed: Pathogen
Actinobacillus succinogenes 130Z	BPrGa_Asuc
Actinomyces sp.	BAcAc_Acti
Actinoplanes missouriensis 431	BAcAc_Amis
Actinoplanes sp. SE50/110	BAcAc_Actp
Actinosynnema mirum DSM 43827	BAcAc_Amir
Acyrthosiphon pisum	EMeAr_Apis
**Advenella kashmirensis WT001**	Removed
Aedes aegypti	Removed: Pathogen
Aequorivita sublithincola DSM 14238	BBaBa_Asub
Aerococcus urinae ACS-120-V-Col10a	BFiBa_Auri
Aeromonas hydrophila subsp. hydrophila ATCC 7966	Removed: Pathogen
Aeromonas salmonicida subsp. salmonicida A449	Removed: Pathogen
Aeromonas veronii B565	BPrGa_Aver
Aeropyrum pernix K1	ACrTh_Aper
	Continued on next page

Table S2 – continued from previous page

Taxa	Abbreviation
Agaricus bisporus var. bisporus	EFuDi_Abis
Aggregatibacter actinomycetemcomitans D7S-1	Removed: Pathogen
Aggregatibacter aphrophilus NJ8700	Removed: Pathogen
Agrobacterium fabrum str. C58	Removed: Pathogen
Agrobacterium radiobacter K84	Removed: Pathogen
Agrobacterium sp. H13-3	BPrAl_Agro
Agrobacterium vitis S4	Removed: Pathogen
Ailuropoda melanoleuca	EOpMe_Amel
Ajellomyces capsulatus NAm1	Removed: Pathogen
Ajellomyces dermatitidis SLH14081	EFuDi_Ader
Akkermansia muciniphila ATCC BAA-835	BChVe_Amuc
Alcanivorax borkumensis SK2	Removed: Pathogen
Alicycliphilus denitrificans K601	BPrBe_Aden
Alicyclobacillus acidocaldarius subsp. acidocaldarius DSM 446	BFiBa_Aaci
Aliivibrio salmonicida LFI1238	Removed: Pathogen
Alistipes finegoldii DSM 17242	BBaBa_Afin
Alkalilimnicola ehrlichii MLHE-1	Removed: Pathogen
Alkaliphilus metalliredigens QYMF	Removed: Pathogen
Alkaliphilus oremlandii OhILAs	Removed: Pathogen
Allochromatium vinosum DSM 180	BPrGa_Avin
Allomyces macrogynus ATCC 38327	EFuBl_Amac
Alternaria brassicicola	EFuDi_Abra
Alteromonas macleodii str. Deep ecotype	BPrGa_Amac
Alteromonas sp. SN2	BPrGa_Alte
Aminobacterium colombiense DSM 12261	BSySy_Acol
Ammonifex degensii KC4	BFiCl_Adeg
Amphimedon queenslandica	EMePo_Aque
Amycolatopsis mediterranei U32	BAcAc_Amed
Amycolicicoccus subflavus DQS3-9A1	BAcAc_Asub
Anabaena variabilis ATCC 29413	BCyNo_Avar
Anaerobaculum mobile DSM 13181	BSySy_Amob
Anaerococcus prevotii DSM 20548	BFiCl_Apre
Anaerolinea thermophila UNI-1	BChAn_Athe
**Anaeromyxobacter dehalogenans 2CP-1**	Removed
Anaeromyxobacter sp. Fw109-5	Removed: Pathogen
Anaplasma centrale str. Israel	Removed: Pathogen
**Anaplasma marginale str. Florida**	Removed
Anaplasma phagocytophilum HZ	Removed: Pathogen
Anolis carolinensis	EOpMe_Acar
Anopheles gambiae	EMeAr_Agam
Anoxybacillus flavithermus WK1	BFiBa_Afla
Apis mellifera	EMeAr_Amel
Aquifex aeolicus VF5	BAqAq_Aaeo
Aquilegia coerulea	EViSt_Acoe
Arabidopsis lyrata	EViSt_Alyr
Arabidopsis thaliana	EViSt_Atha

Continued on next page

Table S2 – continued from previous page

Taxa	Abbreviation
Arcanobacterium haemolyticum DSM 20595	BAcAc_Ahae
Archaeoglobus fulgidus DSM 4304	Removed: Pathogen
Archaeoglobus profundus DSM 5631	AEuAr_Apro
Archaeoglobus veneficus SNP6	AEuAr_Aven
Arcobacter butzleri RM4018	Removed: Pathogen
Arcobacter nitrofigilis DSM 7299	BPrde_Anit
Arcobacter sp. L	BPrde_Arco
Aromatoleum aromaticum EbN1	BPrBe_Aaro
Arthrobacter arilaitensis Re117	BAcAc_Aari
Arthrobacter aurescens TC1	Removed: Pathogen
Arthrobacter chlorophenolicus A6	BAcAc_Achl
Arthrobacter phenanthrenivorans Sphe3	BAcAc_Aphe
Arthrobacter sp. FB24	Removed: Pathogen
Arthroderma benhamiae CBS 112371	EFuDi_Aben
Arthroderma otae CBS 113480	EFuDi_Mcan
Ashbya gossypii ATCC 10895	EFuAs_Agos
Aspergillus carbonarius ITEM 5010	EFuDi_Acar
Aspergillus clavatus NRRL 1	Removed: Pathogen
Aspergillus flavus NRRL3357	Removed: Pathogen
Aspergillus fumigatus Af293	Removed: Pathogen
Aspergillus nidulans FGSC A4	Removed: Pathogen
Aspergillus niger ATCC 1015	EFuDi_Anig
Aspergillus oryzae RIB40	EFuDi_Aory
Aspergillus terreus NIH2624	EFuDi_Ater
Aster yellows witches-broom phytoplasma AYWB	Removed: Pathogen
Asticcacaulis excentricus CB 48	BPrAl_Aexc
Atopobium parvulum DSM 20469	BAcAc_Apar
Atta cephalotes	EMeAr_Acep
Aureococcus anophagefferens	EstPe_Aano
Azoarcus sp. BH72	Removed: Pathogen
Azorhizobium caulinodans ORS 571	BPrAl_Acau
Azospirillum brasilense Sp245	BPrAl_Abra
Azospirillum lipoferum 4B	BPrAl_Alip
Azospirillum sp. B510	BPrAl_Azos
Azotobacter vinelandii DJ	BPrGa_Avie
**Babesia bovis T2Bo**	Removed
Bacillus amyloliquefaciens FZB42	BFiBa_Bamy
Bacillus anthracis str. A0248	Removed: Pathogen
Bacillus atrophaeus 1942	BFiBa_Batr
Bacillus cellulosilyticus DSM 2522	BFiBa_Bcel
Bacillus cereus 03BB102	Removed: Pathogen
Bacillus clausii KSM-K16	BFiBa_Bcla
Bacillus coagulans 2-6	BFiBa_Bcoa
Bacillus halodurans C-125	BFiBa_Bhal
Bacillus licheniformis DSM 13 = ATCC 14580	Removed: Pathogen
Bacillus megaterium DSM 319	BFiBa_Bmeg
	Continued on next page

Table S2 – continued from previous page

Taxa	Abbreviation
Bacillus pseudofirmus OF4	BFiBa_Bpse
Bacillus pumilus SAFR-032	Removed: Pathogen
Bacillus selenitireducens MLS10	BFiBa_Bsel
Bacillus sp. JS	BFiBa_Baci
Bacillus subtilis subsp. subtilis str. 168	BFiBa_Bsub
Bacillus thuringiensis str. Al Hakam	Removed: Pathogen
Bacillus weihenstephanensis KBAB4	Removed: Pathogen
Bacteriovorax marinus SJ	BPrde_Bmar
Bacteroides fragilis YCH46	Removed: Pathogen
Bacteroides helcogenes P 36-108	BBaBa_Bhel
Bacteroides salanitronis DSM 18170	BBaBa_Bsal
Bacteroides thetaiotaomicron VPI-5482	Removed: Pathogen
Bacteroides vulgatus ATCC 8482	Removed: Pathogen
Bartonella bacilliformis KC583	Removed: Pathogen
Bartonella clarridgeiae 73	Removed: Pathogen
Bartonella grahamii as4aup	Removed: Pathogen
Bartonella henselae str. Houston-1	Removed: Pathogen
Bartonella quintana str. Toulouse	Removed: Pathogen
Bartonella tribocorum CIP 105476	Removed: Pathogen
Bathycoccus	EViCh_Bath
Baumannia cicadellinicola str. Hc (Homalodisca coagulata)	Removed: Pathogen
Bdellovibrio bacteriovorus HD100	BPrde_Bbac
Beijerinckia indica subsp. indica ATCC 9039	BPrAl_Bind
Belliella baltica DSM 15883	BBaBa_Bbal
Beutenbergia cavernae DSM 12333	BAcAc_Bcav
Bifidobacterium adolescentis ATCC 15703	Removed: Pathogen
Bifidobacterium animalis subsp. lactis Bl-04	Removed: Pathogen
Bifidobacterium bifidum S17	BAcAc_Bbif
Bifidobacterium breve ACS-071-V-Sch8b	BAcAc_Bbre
Bifidobacterium dentium Bd1	Removed: Pathogen
Bifidobacterium longum subsp. longum JDM301	BAcAc_Blon
Blastococcus saxobsidens DD2	BAcAc_Bsax
**Blattabacterium sp. (Blattella germanica) str. Bge**	Removed
Bombyx mori	EMeAr_Bmor
Bordetella avium 197N	Removed: Pathogen
Bordetella bronchiseptica RB50	Removed: Pathogen
Bordetella parapertussis 12822	Removed: Pathogen
Bordetella pertussis Tohama I	Removed: Pathogen
Bordetella petrii DSM 12804	Removed: Pathogen
Borrelia afzelii PKo	Removed: Pathogen
Borrelia bissettii DN127	Removed: Pathogen
Borrelia burgdorferi B31	Removed: Pathogen
**Borrelia crocidurae str. Achema**	Removed
Borrelia duttonii Ly	Removed: Pathogen
Borrelia garinii PBi	Removed: Pathogen
Borrelia hermsii DAH	Removed: Pathogen

Continued on next page

Table S2 – continued from previous page

Taxa	Abbreviation
Borrelia recurrentis A1	Removed: Pathogen
Borrelia turicatae 91E135	Removed: Pathogen
Bos taurus	EOpMe_Btau
Botryotinia fuckeliana B05.10	Removed: Pathogen
Brachybacterium faecium DSM 4810	BAcAc_Bfae
Brachypodium distachyon	EViSt_Bdis
Brachyspira hyodysenteriae WA1	Removed: Pathogen
Brachyspira intermedia PWS/A	BSpSp_Bint
Brachyspira murdochii DSM 12563	BSpSp_Bmur
Brachyspira pilosicoli 95/1000	BSpSp_Bpil
Bradyrhizobium japonicum USDA 110	BPrAl_Bjap
Bradyrhizobium sp. ORS 278	Removed: Pathogen
Branchiostoma floridae	EMeCh_Bflo
Brevibacillus brevis NBRC 100599	BFiBa_Bbre
Brevundimonas subvibrioides ATCC 15264	BPrAl_Bsub
Brucella abortus S19	Removed: Pathogen
Brucella canis ATCC 23365	Removed: Pathogen
Brucella melitensis ATCC 23457	Removed: Pathogen
Brucella microti CCM 4915	Removed: Pathogen
Brucella ovis ATCC 25840	Removed: Pathogen
Brucella pinnipedialis B2/94	Removed: Pathogen
Brucella suis ATCC 23445	Removed: Pathogen
Brugia malayi	EMeNe_Bmal
Buchnera aphidicola BCc	Removed: Pathogen
Burkholderia ambifaria AMMD	BPrBe_Bamb
Burkholderia cenocepacia HI2424	Removed: Pathogen
Burkholderia gladioli BSR3	BPrBe_Bgla
Burkholderia glumae BGR1	Removed: Pathogen
Burkholderia mallei SAVP1	Removed: Pathogen
Burkholderia multivorans ATCC 17616	BPrBe_Bmul
Burkholderia phymatum STM815	BPrBe_Bphy
Burkholderia phytofirmans PsJN	BPrBe_Bpht
Burkholderia pseudomallei 1106a	Removed: Pathogen
Burkholderia rhizoxinica HKI 454	BPrBe_Brhi
Burkholderia sp. CCGE1002	BPrBe_Burk
Burkholderia thailandensis E264	Removed: Pathogen
Burkholderia vietnamiensis G4	Removed: Pathogen
Burkholderia xenovorans LB400	Removed: Pathogen
Butyrivibrio proteoclasticus B316	BFiCl_Bpro
Caenorhabditis brenneri	EMeNe_Cbre
Caenorhabditis briggsae	EMeNe_Cbri
Caenorhabditis elegans	EOpMe_Cele
Caenorhabditis japonica	EMeNe_Cjap
Caenorhabditis remanei	EMeNe_Crem
Caldicellulosiruptor bescii DSM 6725	BFiCl_Cbes
Caldicellulosiruptor hydrothermalis 108	BFiCl_Chyd
	Continued on next page

Table S2 – continued from previous page

Taxa	Abbreviation
Caldicellulosiruptor kristjanssonii 177R1B	BFiCl_Ckri
Caldicellulosiruptor kronotskyensis 2002	BFiCl_Ckro
Caldicellulosiruptor lactoaceticus 6A	BFiCl_Clac
Caldicellulosiruptor obsidiansis OB47	BFiCl_Cobs
Caldicellulosiruptor owensensis OL	BFiCl_Cowe
Caldicellulosiruptor saccharolyticus DSM 8903	Removed: Pathogen
Caldilinea aerophila DSM 14535 = NBRC 104270	BChCa_Caer
**Caldisericum exile AZM16c01**	Removed
Calditerrivibrio nitroreducens DSM 19672	BDeDe_Cnit
Caldivirga maquilingensis IC-167	ACrTh_Cmaq
Callithrix jacchus	EOpMe_Cjac
Camponotus floridanus	EMeAr_Cflo
Campylobacter concisus 13826	Removed: Pathogen
Campylobacter curvus 525.92	Removed: Pathogen
Campylobacter fetus subsp. fetus 82-40	Removed: Pathogen
Campylobacter hominis ATCC BAA-381	Removed: Pathogen
Campylobacter jejuni subsp. doylei 269.97	Removed: Pathogen
Campylobacter lari RM2100	Removed: Pathogen
Candida albicans SC5314	Removed: Pathogen
Candida dubliniensis CD36	EFuDi_Cdub
Candida glabrata CBS 138	Removed: Pathogen
Candida parapsilosis	EFuDi_Cpar
Candida tropicalis MYA-3404	Removed: Pathogen
Candidatus Accumulibacter phosphatis clade IIA str. UW-1	Removed: Candidatus
Candidatus Amoebophilus asiaticus 5a2	Removed: Candidatus
Candidatus Arthromitus sp. SFB-mouse-Japan	Removed: Candidatus
Candidatus Azobacteroides pseudotrichonymphae genomovar. CFP2	Removed: Candidatus
Candidatus Blochmannia floridanus	Removed: Candidatus
Candidatus Carsonella ruddii PV	Removed: Candidatus
Candidatus Chloracidobacterium thermophilum B	Removed: Candidatus
Candidatus Cloacamonas acidaminovorans	Removed: Candidatus
Candidatus Desulforudis audaxviator MP104C	Removed: Candidatus
Candidatus Hamiltonella defensa 5AT (Acyrthosiphon pisum)	Removed: Candidatus
Candidatus Hodgkinia cicadicola Dsem	Removed: Candidatus
Candidatus Korarchaeum cryptofilum OPF8	Removed: Candidatus
Candidatus Koribacter versatilis Ellin345	Removed: Candidatus
Candidatus Liberibacter asiaticus str. psy62	Removed: Candidatus
Candidatus Methylomirabilis oxyfera	Removed: Candidatus
Candidatus Midichloria mitochondrii IricVA	Removed: Candidatus
Candidatus Moranella endobia PCIT	Removed: Candidatus
Candidatus Nitrospira defluvii	Removed: Candidatus
Candidatus Pelagibacter sp. IMCC9063	Removed: Candidatus
Candidatus Phytoplasma mali	Removed: Candidatus
Candidatus Protochlamydia amoebophila UWE25	Removed: Candidatus
Candidatus Puniceispirillum marinum IMCC1322	Removed: Candidatus
Candidatus Rickettsia amblyommii str. GAT-30V	Removed: Candidatus

Continued on next page

Table S2 – continued from previous page

Taxa	Abbreviation
Candidatus Riesia pediculicola USDA	Removed: Candidatus
Candidatus Ruthia magnifica str. Cm (Calyptogena magnifica)	Removed: Candidatus
Candidatus Solibacter usitatus Ellin6076	Removed: Candidatus
Candidatus Sulcia muelleri DMIN	Removed: Candidatus
Candidatus Sulcia muelleri SMDSEM	Removed: Candidatus
Candidatus Tremblaya princeps PCIT	Removed: Candidatus
Candidatus Vesicomyosocius okutanii HA	Removed: Candidatus
Candidatus Zinderia insecticola CARI	Removed: Candidatus
Canis lupus familiaris	EOpMe_Cfam
Capitella teleta	EMeAn_Capi
Capnocytophaga canimorsus Cc5	Removed: Pathogen
Capnocytophaga ochracea DSM 7271	Removed: Pathogen
Capsaspora owczarzaki	EIcCa_Cowc
Carboxydothermus hydrogenoformans Z-2901	BFiCl_Chyg
Carica papaya	EViSt_Cpap
Carnobacterium sp. 17-4	BFiBa_Carn
Catenulispora acidiphila DSM 44928	BAcAc_Caci
Caulobacter crescentus NA1000	BPrAl_Ccre
Caulobacter segnis ATCC 21756	BPrAl_Cseg
Caulobacter sp. K31	BPrAl_Caul
Cavia porcellus	EOpMe_Cpor
Cellulomonas fimi ATCC 484	BAcAc_Cfim
Cellulomonas flavigena DSM 20109	BAcAc_Cfla
Cellulophaga algicola DSM 14237	BBaBa_Calg
Cellulophaga lytica DSM 7489	BBaBa_Clyt
Cellvibrio japonicus Ueda107	BPrGa_Cjap
Cenarchaeum symbiosum A	AThCe_Csym
Chaetomium globosum CBS 148.51	EFuDi_Cglo
Chelativorans sp. BNC1	Removed: Pathogen
Chitinophaga pinensis DSM 2588	BBaBa_Cpin
Chlamydia muridarum Nigg	Removed: Pathogen
Chlamydia trachomatis B/Jali20/OT	Removed: Pathogen
Chlamydomonas reinhardtii	EViCh_Crei
Chlamydophila abortus S26/3	Removed: Pathogen
Chlamydophila caviae GPIC	Removed: Pathogen
Chlamydophila felis Fe/C-56	Removed: Pathogen
Chlamydophila pecorum E58	Removed: Pathogen
Chlamydophila pneumoniae TW-183	Removed: Pathogen
Chlamydophila psittaci 01DC11	Removed: Pathogen
Chlorella variabilis	EViCh_Chlo
Chlorella vulgaris	EViCh_Cvul
Chlorobaculum parvum NCIB 8327	BBaCh_Cpar
Chlorobium chlorochromatii CaD3	BBaCh_Cchl
Chlorobium limicola DSM 245	BBaCh_Clim
Chlorobium luteolum DSM 273	BBaCh_Clut
Chlorobium phaeobacteroides DSM 266	Removed: Pathogen
	Continued on next page

Table S2 – continued from previous page

Taxa	Abbreviation
Chlorobium phaeovibrioides DSM 265	BBaCh_Cpha
Chlorobium tepidum TLS	BBaCh_Ctep
Chloroflexus aggregans DSM 9485	BChCh_Cagg
Chloroflexus aurantiacus J-10-fl	BChCh_Caur
Chloroflexus sp. Y-400-fl	BChCh_Chlo
Chloroherpeton thalassium ATCC 35110	BBaCh_Ctha
Choloepus hoffmanni	EOpMe_Chof
Chromobacterium violaceum ATCC 12472	Removed: Pathogen
Chromohalobacter salexigens DSM 3043	Removed: Pathogen
Ciona intestinalis	EOpMe_Cint
Ciona savignyi	EOpMe_Csav
Citrobacter koseri ATCC BAA-895	Removed: Pathogen
Citrobacter rodentium ICC168	Removed: Pathogen
Citrus clementina	EViSt_Ccle
Citrus sinensis	EViSt_Csin
Clavibacter michiganensis subsp. michiganensis NCPPB 382	Removed: Pathogen
Clavispora lusitaniae ATCC 42720	Removed: Pathogen
Clostridiales genomosp. BVAB3 str. UPII9-5	BFiCl_Cgen
Clostridium acetobutylicum ATCC 824	BFiCl_Cace
Clostridium beijerinckii NCIMB 8052	Removed: Pathogen
Clostridium botulinum A str. ATCC 3502	Removed: Pathogen
Clostridium cellulolyticum H10	BFiCl_Ccel
Clostridium cellulovorans 743B	BFiCl_Ccev
Clostridium clariflavum DSM 19732	BFiCl_Ccla
Clostridium difficile 630	Removed: Pathogen
Clostridium kluyveri DSM 555	Removed: Pathogen
Clostridium lentocellum DSM 5427	BFiCl_Clen
Clostridium ljungdahlii DSM 13528	BFiCl_Clju
Clostridium novyi NT	Removed: Pathogen
Clostridium perfringens ATCC 13124	Removed: Pathogen
Clostridium phytofermentans ISDg	BFiCl_Cphy
Clostridium saccharolyticum WM1	BFiCl_Csac
Clostridium sp. BNL1100	BFiCl_Clos
Clostridium sticklandii DSM 519	BFiCl_Csti
Clostridium tetani E88	Removed: Pathogen
Clostridium thermocellum ATCC 27405	Removed: Pathogen
Coccidioides immitis RS	Removed: Pathogen
Coccidioides posadasii RMSCC 3488	Removed: Pathogen
Coccomyxa subellipsoidea C-169	EViCh_Cocc
Cochliobolus heterostrophus	EFuDi_Chet
Collimonas fungivorans Ter331	BPrBe_Cfun
Colwellia psychrerythraea 34H	BPrGa_Cpsy
Comamonas testosteroni CNB-2	BPrBe_Ctes
Conexibacter woesei DSM 14684	BAcAc_Cwoe
Coniophora puteana	EFuDi_Cput
Coprinopsis cinerea okayama7#130	Removed: Pathogen

Continued on next page

Table S2 – continued from previous page

Taxa	Abbreviation
Coprothermobacter proteolyticus DSM 5265	BFiCl_Cpro
Coraliomargarita akajimensis DSM 45221	BChVe_Caka
Corallococcus coralloides DSM 2259	BPrde_Ccor
Coriobacterium glomerans PW2	BAcAc_Cglo
Corynebacterium aurimucosum ATCC 700975	BAcAc_Caur
Corynebacterium diphtheriae NCTC 13129	Removed: Pathogen
Corynebacterium efficiens YS-314	BAcAc_Ceff
Corynebacterium glutamicum R	BAcAc_Cglu
Corynebacterium jeikeium K411	Removed: Pathogen
Corynebacterium kroppenstedtii DSM 44385	BAcAc_Ckro
Corynebacterium pseudotuberculosis 316	BAcAc_Cpse
Corynebacterium resistens DSM 45100	BAcAc_Cres
Corynebacterium ulcerans 0102	BAcAc_Culc
Corynebacterium urealyticum DSM 7109	Removed: Pathogen
Corynebacterium variabile DSM 44702	BAcAc_Cvar
Coxiella burnetii Dugway 5J108-111	Removed: Pathogen
Croceibacter atlanticus HTCC2559	BBaBa_Catl
Cronobacter sakazakii ES15	BPrGa_Csak
Cronobacter turicensis z3032	BPrGa_Ctur
Cryphonectria parasitica	EFuDi_Cpas
Cryptobacterium curtum DSM 15641	BAcAc_Ccur
**Cryptosporidium hominis**	Removed
**Cryptosporidium muris**	Removed
Cryptosporidium parvum Iowa II	Removed: Pathogen
Cucumis sativus	EViSt_Csat
Culex quinquefasciatus	EMeAr_Cpip
Cupriavidus metallidurans CH34	BPrBe_Cmet
Cupriavidus necator N-1	BPrBe_Cnec
Cupriavidus taiwanensis LMG 19424	BPrBe_Ctai
Cyanidioschyzon merolae	ERhBa_Cmer
Cyanothece sp. PCC 8802	BCyCh_Cyan
Cyclobacterium marinum DSM 745	BBaBa_Cmar
Cytophaga hutchinsonii ATCC 33406	Removed: Pathogen
Danaus plexippus	EOpMe_Dple
Danio rerio	EOpMe_Drer
Daphnia pulex	EMeAr_Dpul
Dasypus novemcinctus	EOpMe_Dnov
Debaryomyces hansenii CBS767	Removed: Pathogen
Dechloromonas aromatica RCB	BPrBe_Daro
Dechlorosoma suillum PS	BPrBe_Dsui
Deferribacter desulfuricans SSM1	BDeDe_Ddes
Dehalococcoides ethenogenes 195	BChDe_Deth
Dehalococcoides sp. BAV1	BChDe_Deha
Dehalogenimonas lykanthroporepellens BL-DC-9	BChDe_Dlyk
Deinococcus deserti VCD115	BDeDe_Ddee
Deinococcus geothermalis DSM 11300	BDeDe_Dgeo
	Continued on next page

Table S2 – continued from previous page

Taxa	Abbreviation
Deinococcus gobiensis I-0	BDeDe_Dgob
Deinococcus maricopensis DSM 21211	BDeDe_Dmar
Deinococcus proteolyticus MRP	BDeDe_Dpro
Deinococcus radiodurans R1	BDeDe_Drad
Delftia acidovorans SPH-1	BPrBe_Daci
Delftia sp. Cs1-4	BPrBe_Delf
Denitrovibrio acetiphilus DSM 12809	BDeDe_Dace
Desulfarculus baarsii DSM 2075	BPrde_Dbaa
Desulfatibacillum alkenivorans AK-01	BPrde_Dalk
Desulfitobacterium dehalogenans ATCC 51507	BFiCl_Ddeh
Desulfitobacterium hafniense Y51	BFiCl_Dhaf
Desulfobacca acetoxidans DSM 11109	BPrde_Dace
Desulfobacterium autotrophicum HRM2	BPrde_Daut
Desulfobulbus propionicus DSM 2032	BPrde_Dpro
Desulfococcus oleovorans Hxd3	BPrde_Dole
Desulfohalobium retbaense DSM 5692	BPrde_Dret
Desulfomicrobium baculatum DSM 4028	BPrde_Dbac
Desulfomonile tiedjei DSM 6799	BPrde_Dtie
Desulfosporosinus acidiphilus SJ4	BFiCl_Daci
Desulfosporosinus orientis DSM 765	BFiCl_Dori
Desulfotalea psychrophila LSv54	BPrde_Dpsy
Desulfotomaculum acetoxidans DSM 771	BFiCl_Dace
Desulfotomaculum carboxydivorans CO-1-SRB	BFiCl_Dcar
Desulfotomaculum kuznetsovii DSM 6115	BFiCl_Dkuz
Desulfotomaculum reducens MI-1	Removed: Pathogen
Desulfotomaculum ruminis DSM 2154	BFiCl_Drum
Desulfovibrio aespoeensis Aspo-2	BPrde_Daes
Desulfovibrio africanus str. Walvis Bay	BPrde_Dafr
Desulfovibrio alaskensis G20	BPrde_Dala
Desulfovibrio desulfuricans subsp. desulfuricans str. ATCC 27774	BPrde_Ddes
Desulfovibrio magneticus RS-1	BPrde_Dmag
Desulfovibrio salexigens DSM 2638	BPrde_Dsal
Desulfovibrio vulgaris DP4	Removed: Pathogen
Desulfurispirillum indicum S5	BChCh_Dind
Desulfurivibrio alkaliphilus AHT2	BPrde_Dall
Desulfurobacterium thermolithotrophum DSM 11699	BAqAq_Dthe
Desulfurococcus fermentans DSM 16532	ACrTh_Dfer
Desulfurococcus kamchatkensis 1221n	ACrTh_Dkam
Desulfurococcus mucosus DSM 2162	ACrTh_Dmuc
Dichelobacter nodosus VCS1703A	Removed: Pathogen
Dichomitus squalens	EFuDi_Dsqu
Dickeya dadantii Ech703	BPrGa_Ddad
Dickeya zeae Ech1591	BPrGa_Dzea
Dictyoglomus thermophilum H-6-12	BDiDi_Dthe
Dictyoglomus turgidum DSM 6724	BDiDi_Dtur
Dictyostelium discoideum	EMyDi_Ddis
	Continued on next page

Table S2 – continued from previous page

**Taxa**	**Abbreviation**
Dictyostelium purpureum	EMyDi_Dpur
Dinoroseobacter shibae DFL 12	BPrAl_Dshi
Dipodomys ordii	EOpMe_Dord
Dothistroma septosporum NZE10	EFuDi_Dsep
Drosophila ananassae	EMeAr_Dana
Drosophila erecta	EMeAr_Dere
Drosophila grimshawi	EMeAr_Dgri
Drosophila melanogaster	EOpMe_Dmel
Drosophila mojavensis	EMeAr_Dmoj
Drosophila persimilis	EMeAr_Dper
Drosophila pseudoobscura	EMeAr_Dpse
Drosophila sechellia	EMeAr_Dsec
Drosophila simulans	EMeAr_Dsim
Drosophila virilis	EMeAr_Dvir
Drosophila willistoni	EMeAr_Dwil
Drosophila yakuba	EMeAr_Dyak
Dyadobacter fermentans DSM 18053	BBaBa_Dfer
Echinops telfairi	EOpMe_Etel
Ectocarpus siliculosus	EstPX_Esil
Edwardsiella ictaluri 93-146	Removed: Pathogen
Edwardsiella tarda EIB202	Removed: Pathogen
Eggerthella lenta DSM 2243	Removed: Pathogen
Eggerthella sp. YY7918	BAcAc_Egge
Ehrlichia canis str. Jake	Removed: Pathogen
Ehrlichia chaffeensis str. Arkansas	Removed: Pathogen
Ehrlichia ruminantium str. Gardel	Removed: Pathogen
Elusimicrobium minutum Pei191	BElEl_Emin
Emiliania huxleyi	EHaIs_Ehux
**Entamoeba dispar**	Removed
**Entamoeba histolytica**	Removed
**Entamoeba invadens**	Removed
Enterobacter aerogenes KCTC 2190	BPrGa_Eaer
Enterobacter asburiae LF7a	BPrGa_Easb
Enterobacter cloacae subsp. dissolvens SDM	BPrGa_Eclo
Enterobacter sp. 638	BPrGa_Ente
Enterococcus faecalis 62	Removed: Pathogen
Enterococcus faecium Aus0004	Removed: Pathogen
Enterococcus hirae ATCC 9790	BFiBa_Ehir
Equus caballus	EOpMe_Ecab
Erinaceus europaeus	EOpMe_Eeur
Erwinia amylovora ATCC 49946	Removed: Pathogen
Erwinia billingiae Eb661	BPrGa_Ebil
Erwinia pyrifoliae Ep1/96	Removed: Pathogen
Erwinia sp. Ejp617	BPrGa_Erwi
Erwinia tasmaniensis Et1/99	BPrGa_Etas
Erysipelothrix rhusiopathiae str. Fujisawa	BFiEr_Erhu

Continued on next page

Table S2 – continued from previous page

Taxa	Abbreviation
Erythrobacter litoralis HTCC2594	BPrAl_Elit
Escherichia blattae DSM 4481	Removed: Pathogen
Escherichia coli BL21-Gold(DE3)pLysS AG	BPrGa_Ecol
Escherichia fergusonii ATCC 35469	Removed: Pathogen
Ethanoligenens harbinense YUAN-3	BFiCl_Ehar
Eubacterium eligens ATCC 27750	BFiCl_Eeli
Eubacterium limosum KIST612	BFiCl_Elim
Eubacterium rectale ATCC 33656	BFiCl_Erec
Eucalyptus grandis	EViSt_Egra
Exiguobacterium sibiricum 255-15	BFiBa_Esib
Exiguobacterium sp. AT1b	BFiBa_Exig
Felis catus	EOpMe_Fcat
Ferrimonas balearica DSM 9799	BPrGa_Fbal
Ferroglobus placidus DSM 10642	AEuAr_Fpla
Fervidicoccus fontis Kam940	ACrTh_Ffon
Fervidobacterium nodosum Rt17-B1	BThTh_Fnod
Fervidobacterium pennivorans DSM 9078	BThTh_Fpen
Fibrobacter succinogenes subsp. succinogenes S85	BFiFi_Fsuc
Filifactor alocis ATCC 35896	BFiCl_Falo
Finegoldia magna ATCC 29328	Removed: Pathogen
Flavobacteriaceae bacterium 3519-10	BBaBa_Fbac
Flavobacterium branchiophilum FL-15	Removed: Pathogen
Flavobacterium columnare ATCC 49512	Removed: Pathogen
Flavobacterium indicum GPTSA100-9	BBaBa_Find
Flavobacterium johnsoniae UW101	BBaBa_Fjoh
Flavobacterium psychrophilum JIP02/86	Removed: Pathogen
Flexibacter litoralis DSM 6794	BBaBa_Flit
Flexistipes sinusarabici DSM 4947	BDeDe_Fsin
Fluviicola taffensis DSM 16823	BBaBa_Ftaf
Fomitiporia mediterranea MF3/22	EFuDi_Fmed
Fomitopsis pinicola FP-58527 SS1	EFuDi_Fpin
Fragaria vesca	EViSt_Fves
Fragilariopsis cylindrus	EstBa_Fcyl
Francisella cf. novicida 3523	Removed: Pathogen
**Francisella noatunensis subsp. orientalis str. Toba 04**	Removed
Francisella novicida U112	Removed: Pathogen
Francisella philomiragia subsp. philomiragia ATCC 25017	Removed: Pathogen
Francisella sp. TX077308	Removed: Pathogen
Francisella tularensis subsp. tularensis WY96-3418	Removed: Pathogen
Frankia alni ACN14a	Removed: Pathogen
Frankia sp. EAN1pec	Removed: Pathogen
Frankia symbiont of Datisca glomerata	Removed: Pathogen
Frateuria aurantia DSM 6220	BPrGa_Faur
Fusarium oxysporum f. sp. lycopersici 4287	Removed: Pathogen
Fusobacterium nucleatum subsp. nucleatum ATCC 25586	Removed: Pathogen
Gadus morhua	EOpMe_Gmor

Continued on next page

Table S2 – continued from previous page

Taxa	Abbreviation
Gallibacterium anatis UMN179	BPrGa_Gana
Gallionella capsiferriformans ES-2	BPrBe_Gcap
Gallus gallus	EOpMe_Ggal
Gardnerella vaginalis 409-05	BAcAc_Gvag
Gasterosteus aculeatus	EOpMe_Gacu
Gemmatimonas aurantiaca T-27	BGeGe_Gaur
Geobacillus kaustophilus HTA426	BFiBa_Gkau
Geobacillus sp. WCH70	BFiBa_Geob
Geobacillus thermodenitrificans NG80-2	BFiBa_Gthe
Geobacillus thermoglucosidasius C56-YS93	BFiBa_Gthg
Geobacillus thermoleovorans CCB_US3_UF5	BFiBa_Gthl
Geobacter bemidjiensis Bem	BPrde_Gbem
Geobacter daltonii FRC-32	BPrde_Gdal
Geobacter lovleyi SZ	BPrde_Glov
Geobacter metallireducens GS-15	BPrde_Gmet
Geobacter sp. M21	BPrde_Geob
Geobacter sulfurreducens KN400	BPrde_Gsul
Geobacter uraniireducens Rf4	BPrde_Gura
Geodermatophilus obscurus DSM 43160	BAcAc_Gobs
**Giardia lamblia ATCC 50803**	Removed
Gibberella moniliformis 7600	Removed: Pathogen
Gibberella zeae PH-1	Removed: Pathogen
Glaciecola nitratireducens FR1064	BPrGa_Gnit
Glaciecola sp. 4H-3-7+YE-5	BPrGa_Glac
Gloeobacter violaceus PCC 7421	BCyGl_Gvio
Gloeophyllum trabeum ATCC 11539	EFuDi_Gtra
Gluconacetobacter diazotrophicus PAl 5	BPrAl_Gdia
Gluconacetobacter xylinus NBRC 3288	BPrAl_Gxyl
Gluconobacter oxydans 621H	BPrAl_Goxy
Glycine max	EViSt_Gmax
Gordonia bronchialis DSM 43247	Removed: Pathogen
Gordonia polyisoprenivorans VH2	BAcAc_Gpol
Gorilla gorilla	EOpMe_Ggor
Gramella forsetii KT0803	BBaBa_Gfor
Granulibacter bethesdensis CGDNIH1	Removed: Pathogen
Granulicella mallensis MP5ACTX8	BFiAc_Gmal
Granulicella tundricola MP5ACTX9	BFiAc_Gtun
Haemophilus ducreyi 35000HP	Removed: Pathogen
Haemophilus influenzae PittEE	Removed: Pathogen
Haemophilus parainfluenzae T3T1	Removed: Pathogen
Haemophilus parasuis SH0165	Removed: Pathogen
Haemophilus somnus 129PT	Removed: Pathogen
Hahella chejuensis KCTC 2396	BPrGa_Hche
Halalkalicoccus jeotgali B3	AEuHa_Hjeo
Halanaerobium hydrogeniformans	BFiCl_Hhyd
Halanaerobium praevalens DSM 2228	BFiCl_Hpra

Continued on next page

Table S2 – continued from previous page

Taxa	Abbreviation
Haliangium ochraceum DSM 14365	BPrde_Hoch
Haliscomenobacter hydrossis DSM 1100	BBaBa_Hhyd
Haloarcula hispanica ATCC 33960	AEuHa_Hhis
Haloarcula marismortui ATCC 43049	AEuHa_Hmar
Halobacillus halophilus DSM 2266	BFiBa_Hhal
Halobacterium salinarum R1	AEuHa_Hsal
Halobacterium sp. NRC-1	AEuHa_Halo
Haloferax mediterranei ATCC 33500	AEuHa_Hmed
Haloferax volcanii DS2	AEuHa_Hvol
Halogeometricum borinquense DSM 11551	AEuHa_Hbor
Halomicrobium mukohataei DSM 12286	AEuHa_Hmuk
Halomonas elongata DSM 2581	BPrGa_Helo
Halopiger xanaduensis SH-6	AEuHa_Hxan
Haloquadratum walsbyi DSM 16790	Removed: Pathogen
Halorhabdus utahensis DSM 12940	AEuHa_Huta
Halorhodospira halophila SL1	BPrGa_Hhal
Halorubrum lacusprofundi ATCC 49239	AEuHa_Hlac
Haloterrigena turkmenica DSM 5511	AEuHa_Htur
Halothermothrix orenii H 168	BFiCl_Hore
Halothiobacillus neapolitanus c2	BPrGa_Hnea
Harpegnathos saltator	EMeAr_Hsal
Helicobacter acinonychis str. Sheeba	Removed: Pathogen
**Helicobacter bizzozeronii CIII-1**	Removed
**Helicobacter cetorum MIT 00-7128**	Removed
Helicobacter cinaedi PAGU611	BPrde_Hcin
Helicobacter felis ATCC 49179	Removed: Pathogen
Helicobacter hepaticus ATCC 51449	Removed: Pathogen
Helicobacter mustelae 12198	Removed: Pathogen
Helicobacter pylori B38	Removed: Pathogen
Heliconius melpomene	EOpMe_Hmel
Heliconius numata	EOpMe_Hnum
Heliobacterium modesticaldum Ice1	BFiCl_Hmod
Helobdella robusta	EMeAn_Hrob
Herbaspirillum seropedicae SmR1	BPrBe_Hser
Herminiimonas arsenicoxydans	Removed: Pathogen
Herpetosiphon aurantiacus DSM 785	BChCh_Haur
Heterobasidion annosum	EFuDi_Hann
Heterocephalus glaber	EOpMe_Hgla
Hippea maritima DSM 10411	BPrde_Hmar
Hirschia baltica ATCC 49814	BPrAl_Hbal
Homo sapiens	EOpMe_Hsap
Hydra magnipapillata	EMeCn_Hmag
Hydrogenobacter thermophilus TK-6	BAqAq_Hthe
Hydrogenobaculum sp. Y04AAS1	BAqAq_Hydr
Hyperthermus butylicus DSM 5456	ACrTh_Hbut
Hyphomicrobium denitrificans ATCC 51888	BPrAl_Hden
	Continued on next page

Table S2 – continued from previous page

Taxa	Abbreviation
Hyphomicrobium sp. MC1	BPrAl_Hyph
Hyphomonas neptunium ATCC 15444	Removed: Pathogen
Ichthyophthirius multifiliis strain G5	EAlCi_Imul
Idiomarina loihiensis L2TR	BPrGa_Iloi
Ignavibacterium album JCM 16511	BBaIg_Ialb
Ignicoccus hospitalis KIN4/I	ACrTh_Ihos
Ignisphaera aggregans DSM 17230	ACrTh_Iagg
Ilyobacter polytropus DSM 2926	BFuFu_Ipol
Intrasporangium calvum DSM 43043	BAcAc_Ical
Isoptericola variabilis 225	BAcAc_Ivar
Isosphaera pallida ATCC 43644	BPlPl_Ipal
Ixodes scapularis	Removed: Pathogen
Jannaschia sp. CCS1	BPrAl_Jann
Janthinobacterium sp. Marseille	BPrBe_Jant
Jonesia denitrificans DSM 20603	BAcAc_Jden
Kangiella koreensis DSM 16069	BPrGa_Kkor
**Ketogulonicigenium vulgare WSH-001**	Removed
Ketogulonicigenium vulgare Y25	BPrAl_Kvul
Kineococcus radiotolerans SRS30216	Removed: Pathogen
Kitasatospora setae KM-6054	BAcAc_Kset
Klebsiella oxytoca E718	BPrGa_Koxy
Klebsiella pneumoniae subsp. pneumoniae NTUH-K2044	Removed: Pathogen
Klebsiella variicola At-22	BPrGa_Kvar
Kluyveromyces lactis NRRL Y-1140	EFuAs_Klac
Kocuria rhizophila DC2201	BAcAc_Krhi
Komagataella pastoris GS115	EFuDi_Ppas
Kosmotoga olearia TBF 19.5.1	BThTh_Kole
Kribbella flavida DSM 17836	BAcAc_Kfla
Krokinobacter sp. 4H-3-7-5	BBaBa_Krok
Kyrpidia tusciae DSM 2912	BFiBa_Ktus
Kytococcus sedentarius DSM 20547	Removed: Pathogen
Laccaria bicolor S238N-H82	EFuBa_Lbic
Lachancea kluyveri	EFuAs_Lklu
Lachancea thermotolerans CBS 6340	EFuDi_Kthe
Lachancea waltii NCYC 2644	EFuAs_Kwal
Lacinutrix sp. 5H-3-7-4	BBaBa_Laci
Lactobacillus acidophilus 30SC	BFiBa_Laci
Lactobacillus amylovorus GRL1118	BFiBa_Lamy
Lactobacillus brevis ATCC 367	BFiBa_Lbre
Lactobacillus buchneri NRRL B-30929	BFiBa_Lbuc
Lactobacillus casei ATCC 334	BFiBa_Lcas
Lactobacillus crispatus ST1	BFiBa_Lcri
Lactobacillus delbrueckii subsp. bulgaricus ATCC BAA-365	BFiBa_Ldel
Lactobacillus fermentum IFO 3956	BFiBa_Lfer
Lactobacillus gasseri ATCC 33323	BFiBa_Lgas
Lactobacillus helveticus DPC 4571	BFiBa_Lhel

Continued on next page

Table S2 – continued from previous page

Taxa	Abbreviation
Lactobacillus johnsonii FI9785	BFiBa_Ljoh
Lactobacillus kefiranofaciens ZW3	BFiBa_Lkef
Lactobacillus plantarum JDM1	BFiBa_Lpla
Lactobacillus reuteri DSM 20016	BFiBa_Lreu
Lactobacillus rhamnosus Lc 705	Removed: Pathogen
Lactobacillus ruminis ATCC 27782	BFiBa_Lrum
Lactobacillus sakei subsp. sakei 23K	Removed: Pathogen
Lactobacillus salivarius CECT 5713	BFiBa_Lsal
Lactobacillus sanfranciscensis TMW 1.1304	BFiBa_Lsan
Lactococcus garvieae ATCC 49156	Removed: Pathogen
Lactococcus lactis subsp. cremoris MG1363	Removed: Pathogen
Laribacter hongkongensis HLHK9	Removed: Pathogen
Latimeria chalumnae	EOpMe_Lcha
Lawsonia intracellularis PHE/MN1-00	Removed: Pathogen
Leadbetterella byssophila DSM 17132	BBaBa_Lbys
Legionella longbeachae NSW150	Removed: Pathogen
Legionella pneumophila str. Corby	Removed: Pathogen
Leifsonia xyli subsp. xyli str. CTCB07	Removed: Pathogen
Leishmania braziliensis MHOM/BR/75/M2904	Removed: Pathogen
Leishmania infantum JPCM5	Removed: Pathogen
Leishmania major strain Friedlin	Removed: Pathogen
Leishmania mexicana	EEuKi_Lmex
Leptospira biflexa serovar Patoc strain Patoc 1 (Paris)	Removed: Pathogen
Leptospira borgpetersenii serovar Hardjo-bovis str. L550	Removed: Pathogen
Leptospira interrogans serovar Lai str. IPAV	Removed: Pathogen
Leptospirillum ferrooxidans C2-3	BNiNi_Lfer
Leptothrix cholodnii SP-6	BPrBe_Lcho
Leptotrichia buccalis C-1013-b	Removed: Pathogen
Leuconostoc citreum KM20	BFiBa_Lcit
Leuconostoc gasicomitatum LMG 18811	BFiBa_Lgai
Leuconostoc kimchii IMSNU 11154	BFiBa_Lkim
Leuconostoc mesenteroides subsp. mesenteroides ATCC 8293	BFiBa_Lmes
Leuconostoc sp. C2	BFiBa_Leuc
Linepithema humile	EMeAr_Lhum
Listeria innocua Clip11262	Removed: Pathogen
Listeria ivanovii subsp. ivanovii PAM 55	Removed: Pathogen
Listeria monocytogenes serotype 4b str. CLIP 80459	BFiBa_Lmon
Listeria seeligeri serovar 1/2b str. SLCC3954	Removed: Pathogen
Listeria welshimeri serovar 6b str. SLCC5334	Removed: Pathogen
Lodderomyces elongisporus NRRL YB-4239	Removed: Pathogen
Lottia gigantea	EMeMo_Lgig
Loxodonta africana	EOpMe_Lafr
Lysinibacillus sphaericus C3-41	BFiBa_Lsph
Macaca mulatta	EOpMe_Mmul
Macrococcus caseolyticus JCSC5402	BFiBa_Mcas
Macropus eugenii	EOpMe_Meug
	Continued on next page

Table S2 – continued from previous page

Taxa	Abbreviation
Magnaporthe oryzae 70-15	Removed: Pathogen
Magnetococcus marinus MC-1	Removed: Pathogen
Magnetospirillum magneticum AMB-1	BPrAl_Mmag
Mahella australiensis 50-1 BON	BFiCl_Maus
Malassezia globosa CBS 7966	Removed: Pathogen
Malus x domestica	EViSt_Mxxx
Mannheimia succiniciproducens MBEL55E	Removed: Pathogen
Maribacter sp. HTCC2170	BBaBa_Mari
Maricaulis maris MCS10	Removed: Pathogen
Marinithermus hydrothermalis DSM 14884	BDeDe_Mhyd
Marinitoga piezophila KA3	BThTh_Mpie
Marinobacter adhaerens HP15	BPrGa_Madh
Marinobacter aquaeolei VT8	Removed: Pathogen
Marinobacter hydrocarbonoclasticus ATCC 49840	BPrGa_Mhyd
Marinomonas mediterranea MMB-1	BPrGa_Mmed
Marinomonas posidonica IVIA-Po-181	BPrGa_Mpos
Marinomonas sp. MWYL1	BPrGa_Mari
Marivirga tractuosa DSM 4126	BBaBa_Mtra
Medicago truncatula	EViSt_Mtru
Megasphaera elsdenii DSM 20460	Removed: Pathogen
Meiothermus ruber DSM 1279	BDeDe_Mrub
Meiothermus silvanus DSM 9946	BDeDe_Msil
Melampsora larici-populina	EFuDi_Mlar
Meleagris gallopavo	EOpMe_Mgal
Melioribacter roseus P3M	BBaIg_Mros
Melissococcus plutonius DAT561	BFiBa_Mplu
Meloidogyne hapla	EMeNe_Mhap
Meloidogyne incognita	EMeNe_Minc
Mesoplasma florum L1	Removed: Pathogen
Mesorhizobium ciceri biovar biserrulae WSM1271	BPrAl_Mcic
Mesorhizobium loti MAFF303099	BPrAl_Mlot
Mesorhizobium opportunistum WSM2075	BPrAl_Mopp
Mesotoga prima MesG1.Ag.4.2	BThTh_Mpri
Metallosphaera cuprina Ar-4	ACrTh_Mcup
Metallosphaera sedula DSM 5348	Removed: Pathogen
Methanobacterium sp. AL-21	AEuMe_Meth
Methanobrevibacter ruminantium M1	AEuMe_Mrum
Methanobrevibacter smithii ATCC 35061	AEuMe_Msmi
Methanocaldococcus fervens AG86	AEuMe_Mfer
Methanocaldococcus infernus ME	AEuMe_minf
Methanocaldococcus jannaschii DSM 2661	AEuMe_Mjan
Methanocaldococcus sp. FS406-22	AEuMe_Metc
Methanocaldococcus vulcanius M7	AEuMe_Mvul
Methanocella arvoryzae MRE50	Removed: Pathogen
Methanocella conradii HZ254	AEuMe_Mcon
Methanocella paludicola SANAE	AEuMe_Mpal

Continued on next page

Table S2 – continued from previous page

Taxa	Abbreviation
Methanococcoides burtonii DSM 6242	AEuMe_Mbur
Methanococcus aeolicus Nankai-3	AEuMe_Maeo
Methanococcus maripaludis C5	Removed: Pathogen
Methanococcus vannielii SB	Removed: Pathogen
Methanococcus voltae A3	AEuMe_Mvol
Methanocorpusculum labreanum Z	Removed: Pathogen
Methanoculleus bourgensis MS2	AEuMe_Mbou
Methanoculleus marisnigri JR1	Removed: Pathogen
Methanohalobium evestigatum Z-7303	AEuMe_Meve
Methanohalophilus mahii DSM 5219	AEuMe_Mmah
Methanoplanus petrolearius DSM 11571	AEuMe_Mpet
Methanopyrus kandleri AV19	AEuMe_Mkan
Methanoregula boonei 6A8	AEuMe_Mboo
Methanosaeta concilii GP6	AEuMe_Mcoc
Methanosaeta harundinacea 6Ac	AEuMe_Mhar
Methanosaeta thermophila PT	AEuMe_Mthe
Methanosalsum zhilinae DSM 4017	AEuMe_Mzhi
Methanosarcina acetivorans C2A	AEuMe_Mace
Methanosarcina barkeri str. Fusaro	AEuMe_Mbar
Methanosarcina mazei Go1	AEuMe_Mmaz
Methanosphaera stadtmanae DSM 3091	AEuMe_Msta
Methanosphaerula palustris E1-9c	AEuMe_Mpas
Methanospirillum hungatei JF-1	AEuMe_Mhun
Methanothermobacter marburgensis str. Marburg	AEuMe_Mmar
Methanothermobacter thermautotrophicus str. Delta H	AEuMe_Mtha
Methanothermococcus okinawensis IH1	AEuMe_Moki
Methanothermus fervidus DSM 2088	AEuMe_Mfei
Methanotorris igneus Kol 5	AEuMe_Mign
Methylacidiphilum infernorum V4	BChVe_Minf
Methylibium petroleiphilum PM1	BPrBe_Mpet
Methylobacillus flagellatus KT	BPrBe_Mfla
Methylobacterium extorquens AM1	BPrAl_Mext
Methylobacterium extorquens CM4	BPrAl_Mchl
Methylobacterium nodulans ORS 2060	BPrAl_Mnod
Methylobacterium populi BJ001	BPrAl_Mpop
Methylobacterium radiotolerans JCM 2831	BPrAl_Mrad
Methylobacterium sp. 4-46	BPrAl_Meth
Methylocella silvestris BL2	BPrAl_Msil
Methylococcus capsulatus str. Bath	BPrGa_Mcap
Methylomicrobium alcaliphilum	BPrGa_Malc
Methylomonas methanica MC09	BPrGa_Mmet
Methylophaga sp. JAM1	BPrGa_Meth
Methylotenera mobilis JLW8	BPrBe_Mmob
Methylotenera versatilis 301	BPrBe_Mver
Methylovorus glucosetrophus SIP3-4	BPrBe_Mglu
Methylovorus sp. MP688	BPrBe_Meth

Continued on next page

Table S2 – continued from previous page

**Taxa**	**Abbreviation**
Meyerozyma guilliermondii ATCC 6260	Removed: Pathogen
**Micavibrio aeruginosavorus ARL-13**	Removed
Microbacterium testaceum StLB037	BAcAc_Mtes
Microcebus murinus	EOpMe_Mmur
Micrococcus luteus NCTC 2665	BAcAc_Mlut
Microcystis aeruginosa NIES-843	Removed: Pathogen
Microlunatus phosphovorus NM-1	BAcAc_Mpho
Micromonas pusilla CCMP1545	EViCh_Mpus
Micromonas sp. RCC299	EViCh_Micr
Micromonospora aurantiaca ATCC 27029	BAcAc_Maur
Micromonospora sp. L5	BAcAc_Micr
Microsporum gypseum	EFuDi_Mgyp
Mobiluncus curtisii ATCC 43063	BAcAc_Mcur
Modestobacter marinus	BAcAc_Mmar
Monodelphis domestica	EOpMe_Mdom
Monosiga brevicollis	EChCo_Mbre
Moorella thermoacetica ATCC 39073	BFiCl_Mthe
Moraxella catarrhalis RH4	Removed: Pathogen
Mortierella verticillata NRRL 6337	EOpFu_Mver
Mucor circinelloides	EFuFu_Mcir
Muricauda ruestringensis DSM 13258	BBaBa_Mrue
Mus musculus	EOpMe_Mmus
Myceliophthora thermophila ATCC 42464	EFuDi_Sthe
Mycobacterium abscessus ATCC 19977	BAcAc_Mabs
Mycobacterium africanum GM041182	Removed: Pathogen
Mycobacterium avium 104	Removed: Pathogen
Mycobacterium bovis BCG str. Pasteur 1173P2	Removed: Pathogen
Mycobacterium canettii CIPT 140010059	Removed: Pathogen
Mycobacterium chubuense NBB4	BAcAc_Mchu
Mycobacterium gilvum PYR-GCK	BAcAc_Mgil
Mycobacterium intracellulare ATCC 13950	Removed: Pathogen
Mycobacterium leprae Br4923	Removed: Pathogen
Mycobacterium marinum M	Removed: Pathogen
Mycobacterium massiliense str. GO 06	BAcAc_Mmas
Mycobacterium rhodesiae NBB3	BAcAc_Mrho
Mycobacterium smegmatis str. MC2 155	Removed: Pathogen
Mycobacterium sp. JLS	Removed: Pathogen
Mycobacterium tuberculosis KZN 1435	Removed: Pathogen
Mycobacterium ulcerans Agy99	Removed: Pathogen
Mycobacterium vanbaalenii PYR-1	BAcAc_Mvan
Mycoplasma agalactiae PG2	Removed: Pathogen
Mycoplasma arthritidis 158L3-1	Removed: Pathogen
Mycoplasma bovis HB0801	Removed: Pathogen
Mycoplasma capricolum subsp. capricolum ATCC 27343	Removed: Pathogen
Mycoplasma conjunctivae HRC/581	Removed: Pathogen
Mycoplasma crocodyli MP145	Removed: Pathogen

Continued on next page

Table S2 – continued from previous page

Taxa	Abbreviation
Mycoplasma fermentans M64	Removed: Pathogen
Mycoplasma gallisepticum str. F	Removed: Pathogen
Mycoplasma genitalium G37	Removed: Pathogen
**Mycoplasma haemocanis str. Illinois**	Removed
Mycoplasma haemofelis Ohio2	Removed: Pathogen
Mycoplasma hominis ATCC 23114	Removed: Pathogen
Mycoplasma hyopneumoniae 168	Removed: Pathogen
Mycoplasma hyorhinis GDL-1	Removed: Pathogen
Mycoplasma leachii 99/014/6	Removed: Pathogen
Mycoplasma mobile 163K	Removed: Pathogen
Mycoplasma mycoides subsp. capri LC str. 95010	Removed: Pathogen
Mycoplasma penetrans HF-2	Removed: Pathogen
**Mycoplasma pneumoniae 309**	Removed
Mycoplasma pulmonis UAB CTIP	Removed: Pathogen
Mycoplasma putrefaciens KS1	Removed: Pathogen
**Mycoplasma suis KI3806**	Removed
Mycoplasma synoviae 53	Removed: Pathogen
**Mycoplasma wenyonii str. Massachusetts**	Removed
Mycosphaerella populorum SO2202	EFuDi_Smus
Myotis lucifugus	EOpMe_Mluc
Myxococcus fulvus HW-1	BPrde_Mful
Myxococcus xanthus DK 1622	Removed: Pathogen
Naegleria gruberi	EHeSc_Ngru
Nakamurella multipartita DSM 44233	BAcAc_Nmul
**Nanoarchaeum equitans Kin4-M**	Removed
Nasonia vitripennis	EMeAr_Nvit
Natranaerobius thermophilus JW/NM-WN-LF	BFiCl_Nthe
Natrialba magadii ATCC 43099	AEuHa_Nmag
Natrinema sp. J7-2	AEuHa_Natr
Natronomonas pharaonis DSM 2160	AEuHa_Npha
**Nautilia profundicola AmH**	Removed
Nectria haematococca mpVI	EFuDi_Nhae
Neisseria gonorrhoeae NCCP11945	Removed: Pathogen
Neisseria lactamica 020-06	Removed: Pathogen
Neisseria meningitidis FAM18	Removed: Pathogen
Neisseria meningitidis alpha14	Removed: Pathogen
Nematostella vectensis	EMeCn_Nvec
Neorickettsia risticii str. Illinois	Removed: Pathogen
Neorickettsia sennetsu str. Miyayama	Removed: Pathogen
Neosartorya fischeri NRRL 181	Removed: Pathogen
Neospora caninum	EAlAp_Ncan
Neurospora crassa OR74A	EFuAs_Ncra
Neurospora discreta FGSC 8579	EFuDi_Ndis
Neurospora tetrasperma	EFuDi_Ntet
Niastella koreensis GR20-10	BBaBa_Nkor
Nitratifractor salsuginis DSM 16511	BPrde_Nsal

Continued on next page

Table S2 – continued from previous page

Taxa	Abbreviation
Nitratiruptor sp. SB155-2	BPrde_Nitr
Nitrobacter hamburgensis X14	BPrAl_Nham
Nitrobacter winogradskyi Nb-255	BPrAl_Nwin
Nitrosococcus halophilus Nc4	BPrGa_Nhal
Nitrosococcus oceani ATCC 19707	BPrGa_Noce
Nitrosococcus watsonii C-113	BPrGa_Nwat
Nitrosomonas europaea ATCC 19718	BPrBe_Neur
Nitrosomonas eutropha C91	BPrBe_Neut
Nitrosomonas sp. Is79A3	BPrBe_Nitr
Nitrosopumilus maritimus SCM1	AThNi_Nmar
Nitrosospira multiformis ATCC 25196	BPrBe_Nmul
Nocardia cyriacigeorgica GUH-2	BAcAc_Ncyr
Nocardia farcinica IFM 10152	Removed: Pathogen
Nocardioides sp. JS614	Removed: Pathogen
Nocardiopsis dassonvillei subsp. dassonvillei DSM 43111	Removed: Pathogen
Nomascus leucogenys	EOpMe_Nleu
Nostoc azollae 0708	BCyNo_Nazo
Nostoc punctiforme PCC 73102	BCyNo_Npun
Nostoc sp. PCC 7120	BCyNo_Nost
Novosphingobium aromaticivorans DSM 12444	BPrAl_Naro
Novosphingobium sp. PP1Y	BPrAl_Novo
Oceanimonas sp. GK1	BPrGa_Ocea
Oceanithermus profundus DSM 14977	BDeDe_Opro
Oceanobacillus iheyensis HTE831	BFiBa_Oihe
Ochotona princeps	EOpMe_Opri
Ochrobactrum anthropi ATCC 49188	Removed: Pathogen
Odoribacter splanchnicus DSM 20712	BBaBa_Ospl
Oenococcus oeni PSU-1	Removed: Pathogen
Ogataea angusta	EFuDi_Hpol
Oikopleura dioica	EMeCh_Odio
Oligotropha carboxidovorans OM4	BPrAl_Ocar
Olsenella uli DSM 7084	BAcAc_Ouli
Onion yellows phytoplasma OY-M	Removed: Pathogen
Opitutus terrae PB90-1	BChVe_Oter
Oreochromis niloticus	EOpMe_Onil
Orientia tsutsugamushi str. Boryong	Removed: Pathogen
Ornithobacterium rhinotracheale DSM 15997	BBaBa_Orhi
Ornithorhynchus anatinus	EOpMe_Oana
Oryctolagus cuniculus	EOpMe_Ocun
Oryza glaberrima	EViSt_Ogla
Oryza sativa Japonica Group	EViSt_Osat
Oryzias latipes	EOpMe_Olat
Oscillibacter valericigenes Sjm18-20	BFiCl_Oval
Ostreococcus lucimarinus CCE9901	EViCh_Oluc
Ostreococcus sp. RCC809	EViCh_Ostr
Ostreococcus tauri	EViCh_Otau

Continued on next page

Table S2 – continued from previous page

Taxa	Abbreviation
Otolemur garnettii	EOpMe_Ogar
Owenweeksia hongkongensis DSM 17368	BBaBa_Ohon
Paenibacillus mucilaginosus 3016	Removed: Pathogen
Paenibacillus polymyxa M1	Removed: Pathogen
Paenibacillus sp. JDR-2	BFiBa_Paen
Paenibacillus terrae HPL-003	BFiBa_Pter
Paludibacter propionicigenes WB4	BBaBa_Ppro
Pan troglodytes	EOpMe_Ptro
Pantoea ananatis LMG 20103	Removed: Pathogen
Pantoea sp. At-9b	Removed: Pathogen
Pantoea vagans C9-1	Removed: Pathogen
Parabacteroides distasonis ATCC 8503	Removed: Pathogen
Parachlamydia acanthamoebae UV-7	BChCh_Paca
Paracoccidioides brasiliensis Pb18	Removed: Pathogen
Paracoccus denitrificans PD1222	BPrAl_Pden
Paramecium tetraurelia	EAlCi_Ptet
Parvibaculum lavamentivorans DS-1	BPrAl_Plav
Parvularcula bermudensis HTCC2503	BPrAl_Pber
Pasteurella multocida subsp. multocida str. 3480	Removed: Pathogen
Pectobacterium atrosepticum SCRI1043	Removed: Pathogen
Pectobacterium carotovorum subsp. carotovorum PC1	Removed: Pathogen
Pectobacterium wasabiae WPP163	Removed: Pathogen
Pediculus humanus corporis	EMeAr_Phum
Pediococcus claussenii ATCC BAA-344	BFiBa_Pcla
Pediococcus pentosaceus ATCC 25745	Removed: Pathogen
Pedobacter heparinus DSM 2366	BBaBa_Phep
Pedobacter saltans DSM 12145	BBaBa_Psal
Pelagibacterium halotolerans B2	BPrAl_Phal
Pelobacter carbinolicus DSM 2380	BPrde_Pcar
Pelobacter propionicus DSM 2379	Removed: Pathogen
Pelodictyon phaeoclathratiforme BU-1	BBaCh_Ppha
Pelodiscus sinensis	EOpMe_Psin
Pelotomaculum thermopropionicum SI	Removed: Pathogen
Penicillium chrysogenum Wisconsin 54-1255	EFuDi_Pchr
Perkinsus marinus ATCC 50983	Removed: Pathogen
Persephonella marina EX-H1	BAqAq_Pmar
Petromyzon marinus	EOpMe_Pmar
Petrotoga mobilis SJ95	BThTh_Pmob
Phaeobacter gallaeciensis 2.10	BPrAl_Pgal
Phaeodactylum tricornutum CCAP 1055/1	EstBa_Ptri
Phaeosphaeria nodorum SN15	Removed: Pathogen
Phanerochaete chrysosporium RP-78	EFuBa_Pchr
Phenylobacterium zucineum HLK1	BPrAl_Pzuc
Photobacterium profundum SS9	BPrGa_Ppro
**Photorhabdus asymbiotica**	Removed
Photorhabdus luminescens subsp. laumondii TTO1	Removed: Pathogen

Continued on next page

Table S2 – continued from previous page

Taxa	Abbreviation
Phycisphaera mikurensis NBRC 102666	BPlPh_Pmik
Phycomyces blakesleeanus	EFuFu_Pbla
Physcomitrella patens subsp. patens	EViSt_Ppat
Phytophthora capsici	EstOo_Pcap
Phytophthora infestans T30-4	Removed: Pathogen
Phytophthora ramorum	EstOo_Pram
Phytophthora sojae	Removed: Pathogen
Pichia membranifaciens NRRL Y-2026	EFuDi_Pmem
Picrophilus torridus DSM 9790	AEuTh_Ptor
Pirellula staleyi DSM 6068	BPlPl_Psta
Planctomyces brasiliensis DSM 5305	BPlPl_Pbra
Planctomyces limnophilus DSM 3776	BPlPl_Plim
Plasmodium berghei ANKA	Removed: Pathogen
Plasmodium chabaudi	EAlAp_Pcha
Plasmodium falciparum 3D7	Removed: Pathogen
Plasmodium knowlesi strain H	Removed: Pathogen
Plasmodium vivax Sal-1	Removed: Pathogen
Plasmodium yoelii yoelii	EAlAp_Pyoe
Pleurotus ostreatus	EFuDi_Post
Podospora anserina	EFuDi_Pans
Pogonomyrmex barbatus	EMeAr_Pbar
Polaromonas naphthalenivorans CJ2	Removed: Pathogen
Polaromonas sp. JS666	BPrBe_Pola
Polymorphum gilvum SL003B-26A1	BPrAl_Pgil
Polynucleobacter necessarius subsp. asymbioticus QLW-P1DMWA-1	BPrBe_Pnec
Pongo abelii	EOpMe_Pabe
Populus trichocarpa	EViSt_Ptri
Porphyromonas asaccharolytica DSM 20707	BBaBa_Pasa
Porphyromonas gingivalis ATCC 33277	Removed: Pathogen
Postia placenta	EFuDi_Ppla
Prevotella denticola F0289	BBaBa_Pden
Prevotella intermedia 17	Removed: Pathogen
Prevotella melaninogenica ATCC 25845	BBaBa_Pmel
Prevotella ruminicola 23	Removed: Pathogen
Pristionchus pacificus	EMeNe_Ppac
Procavia capensis	EOpMe_Pcap
Prochlorococcus marinus str. MIT 9515	Removed: Pathogen
Propionibacterium acnes SK137	BAcAc_Pacn
Propionibacterium freudenreichii subsp. shermanii CIRM-BIA1	BAcAc_Pfre
Propionibacterium propionicum F0230a	BAcAc_Ppro
Prosthecochloris aestuarii DSM 271	BBaCh_Paes
Proteus mirabilis HI4320	Removed: Pathogen
Providencia stuartii MRSN 2154	BPrGa_Pstu
Pseudoalteromonas atlantica T6c	BPrGa_Patl
Pseudoalteromonas haloplanktis TAC125	BPrGa_Phal
Pseudoalteromonas sp. SM9913	BPrGa_Pseu
	Continued on next page

Table S2 – continued from previous page

Taxa	Abbreviation
Pseudocercospora fijiensis CIRAD86	EFuDi_Mfij
Pseudogulbenkiania sp. NH8B	BPrBe_Pseu
Pseudomonas aeruginosa UCBPP-PA14	Removed: Pathogen
Pseudomonas brassicacearum subsp. brassicacearum NFM421	BPrGa_Pbra
Pseudomonas entomophila L48	Removed: Pathogen
Pseudomonas fluorescens SBW25	Removed: Pathogen
Pseudomonas fulva 12-X	BPrGa_Pful
Pseudomonas mendocina ymp	Removed: Pathogen
Pseudomonas protegens Pf-5	Removed: Pathogen
Pseudomonas putida F1	Removed: Pathogen
Pseudomonas stutzeri A1501	Removed: Pathogen
Pseudomonas syringae pv. tomato str. DC3000	Removed: Pathogen
Pseudonocardia dioxanivorans CB1190	BAcAc_Pdio
Pseudovibrio sp. FO-BEG1	BPrAl_Pseu
Pseudoxanthomonas spadix BD-a59	BPrGa_Pspa
Pseudoxanthomonas suwonensis 11-1	BPrGa_Psuw
Psychrobacter arcticus 273-4	BPrGa_Parc
Psychrobacter cryohalolentis K5	BPrGa_Pcry
Psychrobacter sp. PRwf-1	BPrGa_Psyc
Psychromonas ingrahamii 37	Removed: Pathogen
Pteropus vampyrus	EOpMe_Pvam
Puccinia graminis f. sp. tritici CRL 75-36-700-3	Removed: Pathogen
Punctularia strigosozonata HHB-11173 SS5	EFuDi_Pstr
Pusillimonas sp. T7-7	BPrBe_Pusi
Pyrenophora tritici-repentis	EFuDi_Ptri
Pyrobaculum aerophilum str. IM2	ACrTh_Paer
Pyrobaculum arsenaticum DSM 13514	Removed: Pathogen
Pyrobaculum calidifontis JCM 11548	ACrTh_Pcal
Pyrobaculum islandicum DSM 4184	Removed: Pathogen
Pyrobaculum neutrophilum V24Sta	ACrTh_Tneu
Pyrobaculum oguniense TE7	ACrTh_Pogu
Pyrobaculum sp. 1860	ACrTh_Pyro
Pyrococcus abyssi GE5	AEuTh_Paby
Pyrococcus furiosus COM1	AEuTh_Pfur
Pyrococcus horikoshii OT3	AEuTh_Phor
Pyrococcus sp. ST04	AEuTh_Pyro
Pyrococcus yayanosii CH1	AEuTh_Pyay
Pyrolobus fumarii 1A	ACrTh_Pfum
Pythium ultimum	EStOo_Pult
Rahnella aquatilis CIP 78.65 = ATCC 33071	BPrGa_Raqu
Rahnella sp. Y9602	BPrGa_Rahn
Ralstonia eutropha H16	BPrBe_Reut
Ralstonia eutropha JMP134	BPrBe_ReuJ
Ralstonia pickettii 12D	Removed: Pathogen
Ralstonia solanacearum CFBP2957	Removed: Pathogen
Ramlibacter tataouinensis TTB310	BPrBe_Rtat

Continued on next page

Table S2 – continued from previous page

Taxa	Abbreviation
Rattus norvegicus	EOpMe_Rnor
Renibacterium salmoninarum ATCC 33209	Removed: Pathogen
Rhizobium etli CIAT 652	BPrAl_Retl
Rhizobium leguminosarum bv. viciae 3841	Removed: Pathogen
Rhizopus delemar RA 99-880	Removed: Pathogen
Rhodobacter capsulatus SB 1003	BPrAl_Rcap
Rhodobacter sphaeroides ATCC 17029	Removed: Pathogen
Rhodococcus equi 103S	Removed: Pathogen
Rhodococcus erythropolis PR4	BAcAc_Rery
Rhodococcus jostii RHA1	BAcAc_Rjos
Rhodococcus opacus B4	BAcAc_Ropa
Rhodoferax ferrireducens T118	BPrBe_Rfer
Rhodomicrobium vannielii ATCC 17100	BPrAl_Rvan
Rhodopirellula baltica SH 1	BPlPl_Rbal
Rhodopseudomonas palustris BisA53	Removed: Pathogen
Rhodospirillum centenum SW	BPrAl_Rcen
Rhodospirillum photometricum DSM 122	BPrAl_Rpho
Rhodospirillum rubrum F11	BPrAl_Rrub
Rhodothermus marinus DSM 4252	BBaBa_Rmar
Rhodotorula graminis WP1	EFuDi_Rgra
Rickettsia africae ESF-5	Removed: Pathogen
Rickettsia akari str. Hartford	Removed: Pathogen
**Rickettsia australis str. Cutlack**	Removed
Rickettsia bellii OSU 85-389	Removed: Pathogen
Rickettsia canadensis str. McKiel	Removed: Pathogen
Rickettsia conorii str. Malish 7	Removed: Pathogen
Rickettsia felis URRWXCal2	Removed: Pathogen
**Rickettsia heilongjiangensis 054**	Removed
Rickettsia japonica YH	Removed: Pathogen
Rickettsia massiliae MTU5	Removed: Pathogen
**Rickettsia montanensis str. OSU 85-930**	Removed
**Rickettsia parkeri str. Portsmouth**	Removed
Rickettsia peacockii str. Rustic	Removed: Pathogen
**Rickettsia philipii str. 364D**	Removed
Rickettsia prowazekii str. BuV67-CWPP	BPrAl_Rpro
**Rickettsia rhipicephali str. 3-7-female6-CWPP**	Removed
Rickettsia rickettsii str. Sheila Smith	Removed: Pathogen
Rickettsia slovaca 13-B	Removed: Pathogen
Rickettsia typhi str. Wilmington	Removed: Pathogen
Riemerella anatipestifer ATCC 11845 = DSM 15868	BBaBa_Rana
Robiginitalea biformata HTCC2501	BBaBa_Rbif
Roseburia hominis A2-183	BFiCl_Rhom
Roseiflexus castenholzii DSM 13941	BChCh_Rcas
Roseiflexus sp. RS-1	BChCh_Rose
Roseobacter denitrificans OCh 114	Removed: Pathogen
Roseobacter litoralis Och 149	BPrAl_Rlit

Continued on next page

Table S2 – continued from previous page

Taxa	Abbreviation
Rothia dentocariosa ATCC 17931	BAcAc_Rden
Rothia mucilaginosa DY-18	BAcAc_Rmuc
Rubrivivax gelatinosus IL144	BPrBe_Rgel
Rubrobacter xylanophilus DSM 9941	Removed: Pathogen
Ruegeria pomeroyi DSS-3	BPrAl_Rpom
Ruegeria sp. TM1040	Removed: Pathogen
Ruminococcus albus 7	BFiCl_Ralb
Runella slithyformis DSM 19594	BBaBa_Rsli
Saccharomonospora viridis DSM 43017	Removed: Pathogen
Saccharomyces bayanus	EFuAs_Sbay
Saccharomyces cerevisiae	EOpFu_Scer
Saccharomyces mikatae	EFuAs_Smik
Saccharomyces paradoxus	EFuAs_Spar
Saccharophagus degradans 2-40	BPrGa_Sdeg
Saccharopolyspora erythraea NRRL 2338	BAcAc_Sery
Saccoglossus kowalevskii	EOpMe_Skow
Salinibacter ruber M8	BBaBa_Srub
Salinispora arenicola CNS-205	BAcAc_Sare
Salinispora tropica CNB-440	Removed: Pathogen
Salmonella bongori NCTC 12419	Removed: Pathogen
Salmonella enterica subsp. enterica serovar Paratyphi C strain RKS4594	Removed: Pathogen
Salpingoeca sp. ATCC 50818	EChCo_Prot
Sanguibacter keddieii DSM 10542	BAcAc_Sked
Saprospira grandis str. Lewin	BBaBa_Sgra
Sarcophilus harrisii	EOpMe_Shar
Scheffersomyces stipitis CBS 6054	EFuDi_Psti
Schistosoma mansoni	EMePl_Sman
Schizophyllum commune	EFuDi_Scom
Schizosaccharomyces japonicus yFS275	EFuDi_Sjap
Schizosaccharomyces octosporus yFS286	EFuDi_Soct
Schizosaccharomyces pombe	EFuAs_Spom
Sclerotinia sclerotiorum	EFuDi_Sscl
Sebaldella termitidis ATCC 33386	BFuFu_Ster
Segniliparus rotundus DSM 44985	BAcAc_Srot
Selaginella moellendorffii	EViSt_Smoe
Selenomonas ruminantium subsp. lactilytica TAM6421	Removed: Pathogen
Selenomonas sputigena ATCC 35185	BFiNe_Sspu
Serpula lacrymans var. lacrymans S7.9	EFuDi_Slac
Serratia plymuthica AS9	BPrGa_Sply
Serratia proteamaculans 568	Removed: Pathogen
Serratia sp. AS12	BPrGa_Serr
**Serratia symbiotica str. Cinara cedri**	Removed
Setaria italica	EViSt_Sita
Setosphaeria turcica Et28A	EFuDi_Stur
Shewanella amazonensis SB2B	BPrGa_Sama
	Continued on next page

Table S2 – continued from previous page

Taxa	Abbreviation
Shewanella baltica OS185	Removed: Pathogen
Shewanella denitrificans OS217	BPrGa_Sden
Shewanella frigidimarina NCIMB 400	BPrGa_Sfri
Shewanella halifaxensis HAW-EB4	BPrGa_Shal
Shewanella loihica PV-4	BPrGa_Sloi
Shewanella oneidensis MR-1	Removed: Pathogen
Shewanella pealeana ATCC 700345	BPrGa_Spea
Shewanella piezotolerans WP3	BPrGa_Spie
Shewanella putrefaciens CN-32	Removed: Pathogen
Shewanella sediminis HAW-EB3	BPrGa_Ssed
Shewanella sp. MR-4	BPrGa_Shew
Shewanella violacea DSS12	BPrGa_Svio
Shewanella woodyi ATCC 51908	BPrGa_Swoo
Shigella boydii CDC 3083-94	Removed: Pathogen
Shigella dysenteriae Sd197	Removed: Pathogen
Shigella flexneri 5 str. 8401	Removed: Pathogen
Shigella sonnei Ss046	Removed: Pathogen
Sideroxydans lithotrophicus ES-1	BPrBe_Slit
Simkania negevensis Z	Removed: Pathogen
Sinorhizobium fredii NGR234	BPrAl_Sfre
Sinorhizobium medicae WSM419	Removed: Pathogen
Sinorhizobium meliloti AK83	BPrAl_Smel
Slackia heliotrinireducens DSM 20476	BAcAc_Shel
Sodalis glossinidius str. morsitans	Removed: Pathogen
Solanum lycopersicum	EViSt_Slyc
Solanum tuberosum	EViSt_Stub
Solenopsis invicta	EMeAr_Sinv
Solibacillus silvestris StLB046	BFiBa_Ssil
Solitalea canadensis DSM 3403	BBaBa_Scan
Sorangium cellulosum So ce56	BPrde_Scel
Sorex araneus	EOpMe_Sara
Sorghum bicolor	EViSt_Sbic
Spermophilus tridecemlineatus	EOpMe_Itri
Sphaerobacter thermophilus DSM 20745	BChTh_Sthe
Sphaerochaeta coccoides DSM 17374	BSpSp_Scoc
Sphaerochaeta globus str. Buddy	BSpSp_Sglo
Sphaerochaeta pleomorpha str. Grapes	BSpSp_Sple
Sphaeroforma arctica JP610	EOpOp_Sarc
Sphingobacterium sp. 21	BBaBa_Sphi
Sphingobium chlorophenolicum L-1	BPrAl_Schl
Sphingobium japonicum UT26S	BPrAl_Sjap
Sphingobium sp. SYK-6	BPrAl_Sphi
Sphingomonas wittichii RW1	BPrAl_Swit
Sphingopyxis alaskensis RB2256	BPrAl_Sala
Spirochaeta africana DSM 8902	BSpSp_Safr
Spirochaeta caldaria DSM 7334	BSpSp_Scal

Continued on next page

Table S2 – continued from previous page

Taxa	Abbreviation
Spirochaeta smaragdinae DSM 11293	BSpSp_Ssma
Spirochaeta thermophila DSM 6192	BSpSp_Sthe
Spirosoma linguale DSM 74	BBaBa_Slin
Spizellomyces punctatus DAOM BR117	EFuCh_Spun
Sporobolomyces roseus IAM 13481	EFuDi_Sros
Stackebrandtia nassauensis DSM 44728	BAcAc_Snas
Staphylococcus aureus subsp. aureus JH1	Removed: Pathogen
Staphylococcus carnosus subsp. carnosus TM300	Removed: Pathogen
Staphylococcus epidermidis RP62A	Removed: Pathogen
Staphylococcus haemolyticus JCSC1435	Removed: Pathogen
Staphylococcus lugdunensis HKU09-01	Removed: Pathogen
Staphylococcus pseudintermedius HKU10-03	BFiBa_Spse
Staphylococcus saprophyticus subsp. saprophyticus ATCC 15305	Removed: Pathogen
Staphylothermus hellenicus DSM 12710	ACrTh_Shel
Staphylothermus marinus F1	Removed: Pathogen
Starkeya novella DSM 506	BPrAl_Snov
Stenotrophomonas maltophilia K279a	Removed: Pathogen
Stereum hirsutum FP-91666 SS1	EFuDi_Shir
Stigmatella aurantiaca DW4/3-1	BPrde_Saur
Streptobacillus moniliformis DSM 12112	Removed: Pathogen
Streptococcus agalactiae A909	Removed: Pathogen
Streptococcus dysgalactiae subsp. equisimilis GGS_124	Removed: Pathogen
Streptococcus equi subsp. equi 4047	Removed: Pathogen
Streptococcus gallolyticus UCN34	BFiBa_Sgal
Streptococcus gordonii str. Challis substr. CH1	Removed: Pathogen
Streptococcus infantarius subsp. infantarius CJ18	BFiBa_Sinf
Streptococcus intermedius JTH08	BFiBa_Sint
Streptococcus macedonicus ACA-DC 198	BFiBa_Smac
Streptococcus mitis B6	Removed: Pathogen
Streptococcus mutans NN2025	Removed: Pathogen
Streptococcus oralis Uo5	BFiBa_Sora
Streptococcus parasanguinis ATCC 15912	BFiBa_Spar
Streptococcus parauberis KCTC 11537	BFiBa_Spau
Streptococcus pasteurianus ATCC 43144	BFiBa_Spas
Streptococcus pneumoniae P1031	Removed: Pathogen
Streptococcus pseudopneumoniae IS7493	BFiBa_Spso
Streptococcus pyogenes str. Manfredo	Removed: Pathogen
Streptococcus salivarius 57.I	Removed: Pathogen
Streptococcus sanguinis SK36	Removed: Pathogen
Streptococcus suis P1/7	Removed: Pathogen
Streptococcus thermophilus LMD-9	Removed: Pathogen
Streptococcus uberis 0140J	Removed: Pathogen
Streptomyces avermitilis MA-4680	BAcAc_Save
Streptomyces bingchenggensis BCW-1	BAcAc_Sbin
Streptomyces cattleya NRRL 8057 = DSM 46488	BAcAc_Scat
Streptomyces coelicolor A3(2)	BAcAc_Scoe

Continued on next page

Table S2 – continued from previous page

Taxa	Abbreviation
Streptomyces flavogriseus ATCC 33331	BAcAc_Sfla
Streptomyces griseus subsp. griseus NBRC 13350	BAcAc_Sgri
Streptomyces hygroscopicus subsp. jinggangensis 5008	BAcAc_Shyg
Streptomyces scabiei 87.22	Removed: Pathogen
Streptomyces sp. SirexAA-E	BAcAc_Stre
Streptomyces violaceusniger Tu 4113	BAcAc_Svio
Streptosporangium roseum DSM 43021	BAcAc_Sros
Strongylocentrotus purpuratus	EMeEc_Spur
Sulfobacillus acidophilus DSM 10332	BFiCl_Saci
Sulfolobus acidocaldarius DSM 639	ACrTh_Saci
Sulfolobus islandicus Y.N.15.51	ACrTh_Sisl
Sulfolobus solfataricus P2	ACrTh_Ssol
Sulfolobus tokodaii str. 7	ACrTh_Stok
Sulfuricurvum kujiense DSM 16994	BPrde_Skuj
Sulfurihydrogenibium azorense Az-Fu1	BAqAq_Sazo
Sulfurihydrogenibium sp. YO3AOP1	BAqAq_Sulf
Sulfurimonas autotrophica DSM 16294	BPrde_Saut
Sulfurimonas denitrificans DSM 1251	BPrde_Sden
Sulfurospirillum barnesii SES-3	BPrde_Sbar
Sulfurospirillum deleyianum DSM 6946	BPrde_Sdel
Sulfurovum sp. NBC37-1	Removed: Pathogen
Sus scrofa	EOpMe_Sscr
Symbiobacterium thermophilum IAM 14863	BFiCl_Sthe
Synechococcus elongatus PCC 7942	BCyCh_Selo
Synechococcus sp. CC9311	Removed: Pathogen
Synechocystis sp. PCC 6803	BCyCh_Syne
Syntrophobacter fumaroxidans MPOB	Removed: Pathogen
Syntrophobotulus glycolicus DSM 8271	BFiCl_Sgly
Syntrophomonas wolfei subsp. wolfei str. Goettingen	BFiCl_Swol
Syntrophothermus lipocalidus DSM 12680	BFiCl_Slip
Syntrophus aciditrophicus SB	BPrde_Saci
Taeniopygia guttata	EOpMe_Tgut
Takifugu rubripes	EOpMe_Trub
Talaromyces marneffei ATCC 18224	Removed: Pathogen
Tannerella forsythia ATCC 43037	Removed: Pathogen
Tarsius syrichta	EOpMe_Tsyr
**Taylorella asinigenitalis MCE3**	Removed
**Taylorella equigenitalis MCE9**	Removed
Tepidanaerobacter acetatoxydans Re1	BFiCl_Tace
Teredinibacter turnerae T7901	BPrGa_Ttur
Terriglobus roseus DSM 18391	BFiAc_Tros
Terriglobus saanensis SP1PR4	BFiAc_Tsaa
Tetragenococcus halophilus NBRC 12172	BFiBa_Thal
Tetrahymena thermophila SB210	EAlCi_Tthe
Tetranychus urticae	EOpMe_Turt
Tetraodon nigroviridis	EOpMe_Tnig

Continued on next page

Table S2 – continued from previous page

**Taxa**	**Abbreviation**
Thalassiosira pseudonana CCMP1335	EstBa_Tpse
Thauera sp. MZ1T	BPrBe_Thau
Thecamonas trahens ATCC 50062	EApAp_Ttra
**Theileria annulata**	Removed
**Theileria parva**	Removed
Theobroma cacao	EViSt_Tcac
Thermaerobacter marianensis DSM 12885	BFiCl_Tmar
Thermanaerovibrio acidaminovorans DSM 6589	BSySy_Taci
Thermincola potens JR	BFiCl_Tpot
Thermoanaerobacter brockii subsp. finnii Ako-1	BFiCl_Tbro
Thermoanaerobacter italicus Ab9	BFiCl_Tita
Thermoanaerobacter mathranii subsp. mathranii str. A3	BFiCl_Tmat
Thermoanaerobacter pseudethanolicus ATCC 33223	BFiCl_Tpse
Thermoanaerobacter sp. X514	BFiCl_Ther
Thermoanaerobacter tengcongensis MB4	BFiCl_Tten
Thermoanaerobacter wiegelii Rt8.B1	BFiCl_Twie
Thermoanaerobacterium thermosaccharolyticum DSM 571	BFiCl_Tthe
Thermoanaerobacterium xylanolyticum LX-11	BFiCl_Txyl
Thermobaculum terrenum ATCC BAA-798	BunTh_Tter
Thermobifida fusca YX	Removed: Pathogen
Thermobispora bispora DSM 43833	BAcAc_Tbis
Thermococcus barophilus MP	AEuTh_Tbar
Thermococcus gammatolerans EJ3	AEuTh_Tgam
Thermococcus kodakarensis KOD1	AEuTh_Tkod
Thermococcus onnurineus NA1	AEuTh_Tonn
Thermococcus sibiricus MM 739	AEuTh_Tsib
Thermococcus sp. 4557	AEuTh_Ther
Thermocrinis albus DSM 14484	BAqAq_Talb
Thermodesulfatator indicus DSM 15286	BThTh_Tind
Thermodesulfobacterium geofontis OPF15	BThTh_Ther
Thermodesulfobium narugense DSM 14796	BFiCl_Tnar
Thermodesulfovibrio yellowstonii DSM 11347	BNiNi_Tyel
Thermofilum pendens Hrk 5	Removed: Pathogen
Thermogladius cellulolyticus 1633	ACrTh_Tcel
Thermomicrobium roseum DSM 5159	BChTh_Tros
Thermomonospora curvata DSM 43183	BAcAc_Tcur
Thermoplasma acidophilum DSM 1728	AEuTh_Taci
Thermoplasma volcanium GSS1	AEuTh_Tvol
Thermoproteus tenax Kra 1	ACrTh_Tten
Thermoproteus uzoniensis 768-20	ACrTh_Tuzo
Thermosediminibacter oceani DSM 16646	BFiCl_Toce
Thermosipho africanus TCF52B	BThTh_Tafr
Thermosipho melanesiensis BI429	BThTh_Tmel
Thermosphaera aggregans DSM 11486	ACrTh_Tagg
Thermosynechococcus elongatus BP-1	BCyCh_Telo
Thermotoga lettingae TMO	BThTh_Tlet

Continued on next page

Table S2 – continued from previous page

Taxa	Abbreviation
Thermotoga maritima MSB8	BThTh_Tmar
Thermotoga naphthophila RKU-10	BThTh_Tnap
Thermotoga neapolitana DSM 4359	BThTh_Tnea
Thermotoga petrophila RKU-1	Removed: Pathogen
Thermotoga sp. RQ2	BThTh_Thet
Thermotoga thermarum DSM 5069	BThTh_Tthe
Thermovibrio ammonificans HB-1	BAqAq_Tamm
Thermovirga lienii DSM 17291	BSySy_Tlie
Thermus scotoductus SA-01	BDeDe_Tsco
Thermus sp. CCB_US3_UF1	BDeDe_Ther
Thermus thermophilus HB27	BDeDe_Tthe
Thielavia terrestris NRRL 8126	EFuDi_Tter
Thioalkalimicrobium cyclicum ALM1	BPrGa_Tcyc
Thioalkalivibrio sp. K90mix	BPrGa_Thio
Thioalkalivibrio sulfidophilus HL-EbGr7	BPrGa_Tsul
Thiobacillus denitrificans ATCC 25259	BPrBe_Tden
Thiocystis violascens DSM 198	BPrGa_Tvio
Thiomicrospira crunogena XCL-2	BPrGa_Tcru
Thiomonas intermedia K12	BPrBe_Tint
Tistrella mobilis KA081020-065	BPrAl_Tmob
Tolumonas auensis DSM 9187	BPrGa_Taue
Toxoplasma gondii GT1	EAlAp_Tgon
Toxoplasma gondii ME49	EAlAp_TgoM
Toxoplasma gondii VEG	EAlAp_TgoV
Trametes versicolor FP-101664 SS1	EFuDi_Tver
Tremella mesenterica	EFuDi_Tmes
Treponema azotonutricium ZAS-9	BSpSp_Tazo
Treponema brennaborense DSM 12168	BSpSp_Tbre
Treponema denticola ATCC 35405	Removed: Pathogen
Treponema pallidum subsp. pallidum SS14	Removed: Pathogen
Treponema paraluiscuniculi Cuniculi A	Removed: Pathogen
Treponema primitia ZAS-2	BSpSp_Tpri
Treponema succinifaciens DSM 2489	BSpSp_Tsuc
Tribolium castaneum	EMeAr_Tcas
Trichoderma atroviride	EFuDi_Tatr
Trichoderma reesei QM6a	EFuAs_Tree
Trichoderma virens Gv29-8	EFuDi_Tvir
Trichodesmium erythraeum IMS101	Removed: Pathogen
Trichomonas vaginalis	EPaTr_Tvag
Trichophyton equinum CBS 127.97	EFuDi_Tequ
Trichophyton rubrum CBS 118892	EFuDi_Trub
Trichophyton tonsurans CBS 112818	EFuDi_Tton
Trichophyton verrucosum HKI 0517	EFuDi_Tveu
Trichoplax adhaerens	EMePl_Tadh
Tropheryma whipplei TW08/27	Removed: Pathogen
Truepera radiovictrix DSM 17093	BDeDe_Trad

Continued on next page

Table S2 – continued from previous page

Taxa	Abbreviation
Trypanosoma brucei TREU927	Removed: Pathogen
Trypanosoma brucei gambiense	EEuKi_Tbru
Trypanosoma congolense	EEuKi_Tcon
Trypanosoma cruzi strain CL Brener	Removed: Pathogen
Trypanosoma vivax	EEuKi_Tviv
Tsukamurella paurometabola DSM 20162	BAcAc_Tpau
Tuber melanosporum	EFuDi_Tmel
Tupaia belangeri	EOpMe_Tbel
Turneriella parva DSM 21527	BSpSp_Tpar
Tursiops truncatus	EOpMe_Ttru
Uncinocarpus reesii 1704	Removed: Pathogen
Ureaplasma parvum serovar 3 str. ATCC 27815	Removed: Pathogen
Ureaplasma urealyticum serovar 10 str. ATCC 33699	Removed: Pathogen
Ustilago maydis	EFuBa_Umay
Vanderwaltozyma polyspora DSM 70294	EFuDi_Vpol
Variovorax paradoxus S110	BPrBe_Vpar
Veillonella parvula DSM 2008	Removed: Pathogen
Verminephrobacter eiseniae EF01-2	Removed: Pathogen
Verrucosispora maris AB-18-032	BAcAc_Vmar
Verticillium albo-atrum VaMs.102	Removed: Pathogen
Verticillium dahliae VdLs.17	Removed: Pathogen
Vibrio anguillarum 775	BPrGa_Vang
Vibrio cholerae O395	Removed: Pathogen
Vibrio fischeri MJ11	BPrGa_Vfis
Vibrio furnissii NCTC 11218	BPrGa_Vfur
Vibrio harveyi ATCC BAA-1116	Removed: Pathogen
Vibrio parahaemolyticus RIMD 2210633	Removed: Pathogen
Vibrio sp. Ex25	BPrGa_Vibr
Vibrio splendidus LGP32	Removed: Pathogen
Vibrio vulnificus MO6-24/O	Removed: Pathogen
Vicugna pacos	EOpMe_Vpac
Vitis vinifera	EViSt_Vvin
Volvox carteri f. nagariensis	EViCh_Vcar
Vulcanisaeta distributa DSM 14429	ACrTh_Vdis
Vulcanisaeta moutnovskia 768-28	ACrTh_Vmou
Waddlia chondrophila WSU 86-1044	BChCh_Wcho
Wallemia sebi CBS 633.66	EFuDi_Wseb
Weeksella virosa DSM 16922	BBaBa_Wvir
Weissella koreensis KACC 15510	BFiBa_Wkor
Wickerhamomyces anomalus	EFuDi_Wano
**Wigglesworthia glossinidia endosymbiont of Glossina brevipalpis**	Removed
**Wolbachia endosymbiont of Culex quinquefasciatus Pel**	Removed
**Wolbachia sp. wRi**	Removed
Wolfiporia cocos MD-104 SS10	EFuDi_Wcoc
Wolinella succinogenes DSM 1740	Removed: Pathogen

Continued on next page

Table S2 – continued from previous page

**Taxa**	**Abbreviation**
Xanthobacter autotrophicus Py2	BPrAl_Xaut
Xanthomonas albilineans GPE PC73	Removed: Pathogen
Xanthomonas axonopodis pv. citri str. 306	Removed: Pathogen
Xanthomonas campestris pv. campestris str. B100	Removed: Pathogen
Xanthomonas oryzae pv. oryzae PXO99A	Removed: Pathogen
Xenopus (Silurana) tropicalis	EOpMe_Xtro
Xenopus laevis	EMeCh_Xlae
Xenorhabdus bovienii SS-2004	Removed: Pathogen
Xenorhabdus nematophila ATCC 19061	Removed: Pathogen
Xylanimonas cellulosilytica DSM 15894	BAcAc_Xcel
Xylella fastidiosa M23	Removed: Pathogen
Yarrowia lipolytica CLIB122	EFuAs_Ylip
Yersinia enterocolitica subsp. enterocolitica 8081	Removed: Pathogen
Yersinia pestis Pestoides F	Removed: Pathogen
Yersinia pseudotuberculosis IP 31758	Removed: Pathogen
Zea mays subsp. mays	EViSt_Zmay
Zobellia galactanivorans	BBaBa_Zgal
Zunongwangia profunda SM-A87	BBaBa_Zpro
Zygosaccharomyces rouxii	EFuDi_Zrou
Zymomonas mobilis subsp. mobilis NCIMB 11163	BPrAl_Zmob
Zymoseptoria tritici IPO323	Removed: Pathogen
Cellvibrio gilvus ATCC 13127	BAcAc_Cgil
cyanobacterium UCYN-A	BCyCh_cUCY
gamma proteobacterium HdN1	BPrGa_gpro
halophilic archaeon DL31	Aunun_harc
**secondary endosymbiont of Ctenarytaina eucalypti**	Removed
uncultured Termite group 1 bacterium phylotype Rs-D17	BElen_uTer
