# Supplementary material for: Exploring Fold Space Preferences of New-born and Ancient Protein Superfamilies
Source: PLoS Comput Biol. 2013 Nov 14;9(11):e1003325. doi: 10.1371/journal.pcbi.1003325 (PMC3828129; doi:10.1371/journal.pcbi.1003325)
Supplement: Table S3 — ASTRAL40 domains. The number of domains for each superfamily with representative structures in the ASTRAL40 set. (PDF) [file pcbi.1003325.s008.pdf]

Table S3. ASTRAL40 domains.

sf	freq	sf	freq	sf	freq	sf	freq
a.1.1	25	a.18.1	1	a.30.6	1	a.69.2	2
a.1.2	2	a.19.1	2	a.31.1	1	a.69.3	1
a.2.1	1	a.20.1	2	a.32.1	2	a.69.4	2
a.2.3	1	a.22.1	12	a.34.1	2	a.70.2	1
a.2.7	1	a.23.1	1	a.34.2	1	a.71.1	1
a.2.10	1	a.23.2	1	a.34.4	1	a.72.1	1
a.2.11	4	a.23.3	1	a.35.1	20	a.73.1	3
a.2.13	1	a.23.7	1	a.36.1	1	a.74.1	8
a.2.16	1	a.24.1	1	a.38.1	3	a.75.1	2
a.2.17	2	a.24.2	1	a.39.1	23	a.76.1	2
a.2.18	1	a.24.3	6	a.39.2	4	a.77.1	3
a.2.19	2	a.24.4	1	a.39.3	2	a.78.1	1
a.3.1	25	a.24.7	1	a.40.1	4	a.79.1	3
a.4.1	39	a.24.8	1	a.42.1	1	a.80.1	2
a.4.2	1	a.24.9	1	a.43.1	8	a.83.1	2
a.4.5	67	a.24.10	6	a.45.1	17	a.85.1	1
a.4.6	6	a.24.11	1	a.46.2	2	a.86.1	1
a.4.12	1	a.24.12	1	a.46.3	1	a.87.1	1
a.4.13	3	a.24.13	4	a.47.2	3	a.89.1	2
a.5.2	3	a.24.15	2	a.47.5	1	a.90.1	1
a.5.3	2	a.24.16	1	a.48.1	1	a.91.1	3
a.5.7	3	a.24.17	1	a.48.2	1	a.92.1	1
a.5.8	1	a.24.20	1	a.48.4	1	a.93.1	8
a.5.10	1	a.24.22	1	a.48.5	1	a.96.1	8
a.6.1	5	a.24.26	3	a.51.1	1	a.97.1	1
a.7.1	4	a.24.27	1	a.52.1	3	a.99.1	3
a.7.2	1	a.24.28	1	a.53.1	1	a.100.1	18
a.7.3	2	a.25.1	29	a.55.1	3	a.101.1	3
a.7.5	1	a.25.2	2	a.56.1	3	a.102.1	19
a.7.7	1	a.25.6	1	a.60.1	6	a.102.2	4
a.7.8	2	a.26.1	10	a.60.2	2	a.102.3	6
a.7.12	2	a.27.1	3	a.60.3	1	a.102.4	8
a.7.17	1	a.28.1	2	a.60.4	3	a.102.5	1
a.8.1	3	a.28.2	1	a.60.5	1	a.103.1	2
a.8.2	1	a.28.3	1	a.60.6	2	a.104.1	12
a.8.3	2	a.29.2	5	a.60.7	1	a.108.1	1
a.8.4	2	a.29.3	7	a.60.8	3	a.110.1	1
a.8.5	1	a.29.6	2	a.60.12	2	a.111.1	4
a.8.10	1	a.29.7	1	a.60.13	1	a.114.1	1
a.8.11	1	a.29.8	1	a.60.14	1	a.115.1	1
a.9.1	1	a.29.9	1	a.60.15	1	a.116.1	2
a.10.1	1	a.29.11	1	a.61.1	1	a.117.1	1
a.11.1	2	a.29.13	2	a.64.1	1	a.118.1	17
a.11.2	2	a.29.14	2	a.64.2	1	a.118.3	3
a.13.1	1	a.29.16	1	a.65.1	1	a.118.4	1

Continued on next page

Table S3 – continued from previous page

sf	freq	sf	freq	sf	freq	sf	freq
a.14.1	1	a.30.1	1	a.66.1	1	a.118.5	1
a.16.1	3	a.30.4	1	a.69.1	2	a.118.6	1
a.118.7	3	a.158.1	1	a.248.1	1	b.1.24	1
a.118.8	17	a.159.1	1	a.254.1	1	b.1.26	1
a.118.9	3	a.159.3	1	a.255.1	1	b.1.28	2
a.118.11	1	a.159.5	1	a.257.1	1	b.2.1	1
a.118.12	1	a.160.1	4	a.258.1	1	b.2.2	8
a.118.15	1	a.161.1	1	a.259.1	1	b.2.3	14
a.118.18	2	a.174.1	2	a.261.1	1	b.2.4	1
a.118.21	1	a.176.1	1	a.265.1	1	b.2.5	5
a.118.23	1	a.177.1	1	a.266.1	1	b.2.6	1
a.118.25	1	a.180.1	1	a.268.1	1	b.3.1	7
a.119.1	1	a.182.1	1	a.270.1	1	b.3.2	1
a.120.1	1	a.184.1	1	a.271.1	1	b.3.3	1
a.121.1	20	a.185.1	1	a.273.1	1	b.3.4	1
a.123.1	11	a.186.1	2	a.275.1	1	b.3.5	1
a.124.1	3	a.187.1	1	a.276.1	1	b.3.6	3
a.126.1	6	a.189.1	1	a.277.1	1	b.5.1	1
a.127.1	6	a.192.1	1	a.278.1	4	b.6.1	32
a.128.1	2	a.193.1	1	a.279.1	1	b.6.2	2
a.129.1	1	a.194.1	2	a.280.1	1	b.7.1	8
a.130.1	6	a.195.1	1	a.281.1	2	b.7.2	2
a.131.1	1	a.196.1	1	a.283.1	1	b.7.3	1
a.132.1	8	a.198.1	1	a.286.1	1	b.7.5	1
a.133.1	4	a.199.1	1	a.287.1	2	b.8.1	2
a.134.1	1	a.204.1	4	a.288.1	1	b.11.1	5
a.135.1	1	a.206.1	1	a.289.1	1	b.12.1	3
a.136.1	1	a.211.1	11	a.292.1	1	b.16.1	1
a.137.2	1	a.213.1	7	a.296.1	1	b.17.1	3
a.137.4	1	a.214.1	1	b.1.1	62	b.18.1	40
a.137.5	1	a.216.1	1	b.1.2	12	b.19.1	2
a.137.7	1	a.219.1	1	b.1.4	10	b.21.1	5
a.137.9	1	a.220.1	1	b.1.5	4	b.22.1	10
a.137.15	1	a.221.1	1	b.1.6	2	b.23.1	3
a.138.1	15	a.222.1	1	b.1.7	1	b.23.2	1
a.139.1	2	a.224.1	1	b.1.8	4	b.24.1	5
a.140.2	1	a.226.1	1	b.1.9	2	b.25.1	1
a.140.3	1	a.228.1	1	b.1.10	4	b.26.1	6
a.140.4	1	a.230.1	1	b.1.11	2	b.27.1	1
a.141.1	1	a.233.1	1	b.1.13	2	b.29.1	47
a.142.1	2	a.237.1	1	b.1.14	2	b.30.2	4
a.144.1	1	a.241.1	1	b.1.16	1	b.30.5	17
a.145.1	1	a.243.1	2	b.1.17	1	b.31.1	2
a.146.1	1	a.244.1	1	b.1.18	42	b.33.1	12
a.149.1	4	a.245.1	1	b.1.19	1	b.34.1	3
a.152.1	8	a.246.1	1	b.1.20	2	b.34.2	14

Continued on next page

Table S3 – continued from previous page

sf	freq	sf	freq	sf	freq	sf	freq
a.156.1	5	a.246.2	2	b.1.21	1	b.34.3	2
a.157.1	1	a.247.1	1	b.1.22	1	b.34.4	4
b.34.5	4	b.53.1	1	b.76.1	1	b.115.1	1
b.34.6	4	b.55.1	25	b.76.2	1	b.117.1	1
b.34.8	1	b.56.1	2	b.77.3	4	b.120.1	1
b.34.9	10	b.57.1	2	b.78.1	2	b.121.1	4
b.34.10	1	b.58.1	2	b.80.1	17	b.121.2	2
b.34.11	2	b.60.1	28	b.80.2	1	b.121.3	2
b.34.12	1	b.61.1	2	b.80.6	1	b.121.4	3
b.34.13	5	b.61.2	2	b.80.7	1	b.121.6	1
b.34.15	1	b.61.3	2	b.80.8	3	b.121.7	2
b.34.16	1	b.61.4	1	b.81.1	11	b.122.1	19
b.35.1	12	b.61.5	1	b.81.3	1	b.123.1	1
b.36.1	15	b.61.6	1	b.82.1	38	b.125.1	3
b.37.1	2	b.62.1	4	b.82.2	11	b.127.1	1
b.38.1	8	b.63.1	1	b.82.3	7	b.128.1	1
b.38.5	1	b.64.1	5	b.82.4	1	b.129.1	1
b.39.1	1	b.65.1	2	b.82.5	2	b.129.2	1
b.40.1	1	b.66.1	1	b.82.6	2	b.130.1	1
b.40.2	11	b.67.1	1	b.83.1	1	b.131.1	1
b.40.3	2	b.67.2	8	b.84.1	1	b.132.1	1
b.40.4	34	b.67.3	1	b.84.2	6	b.133.1	1
b.40.5	2	b.68.1	11	b.84.3	2	b.134.1	1
b.40.6	8	b.68.2	1	b.84.4	1	b.135.1	1
b.40.10	1	b.68.3	1	b.85.1	2	b.136.1	1
b.40.11	1	b.68.4	1	b.85.2	1	b.137.1	2
b.40.14	2	b.68.5	1	b.85.3	1	b.138.1	1
b.41.1	1	b.68.6	4	b.85.4	8	b.139.1	1
b.42.1	6	b.68.8	1	b.85.6	1	b.141.1	1
b.42.2	12	b.68.9	2	b.85.7	1	b.143.1	1
b.42.4	7	b.68.10	2	b.86.1	3	b.145.1	1
b.42.8	1	b.68.11	1	b.87.1	3	b.148.1	1
b.43.3	6	b.69.1	1	b.88.1	4	b.149.1	1
b.43.4	10	b.69.2	2	b.89.1	1	b.150.1	1
b.43.5	3	b.69.3	1	b.91.1	1	b.152.1	1
b.44.1	3	b.69.4	5	b.92.1	16	b.154.1	1
b.44.2	5	b.69.5	1	b.93.1	1	b.156.1	1
b.45.1	20	b.69.7	2	b.95.1	1	b.157.1	1
b.45.2	2	b.69.11	1	b.97.1	2	b.158.1	1
b.46.1	3	b.70.1	2	b.98.1	1	b.159.1	1
b.47.1	31	b.70.2	1	b.100.1	3	b.159.2	2
b.49.1	2	b.70.3	1	b.103.1	1	b.161.1	1
b.49.2	5	b.71.1	27	b.105.1	2	b.163.1	2
b.49.3	3	b.72.1	3	b.107.1	2	b.167.1	1
b.50.1	15	b.72.2	1	b.108.1	5	b.168.1	1
b.51.1	3	b.72.3	1	b.109.1	2	b.169.1	3

Continued on next page

Table S3 – continued from previous page

sf	freq	sf	freq	sf	freq	sf	freq
b.52.1	4	b.74.1	3	b.111.1	1	b.171.1	1
b.52.2	10	b.75.1	1	b.113.1	5	b.172.1	2
b.174.1	1	c.8.5	2	c.38.1	1	c.67.2	1
b.175.1	1	c.8.6	1	c.39.1	2	c.67.3	1
b.176.1	1	c.8.7	1	c.40.1	1	c.68.1	18
b.178.1	1	c.8.8	1	c.41.1	8	c.69.1	64
c.1.1	1	c.8.9	1	c.42.1	7	c.70.1	2
c.1.2	17	c.8.10	1	c.43.1	5	c.71.1	10
c.1.3	2	c.9.1	1	c.44.1	2	c.72.1	15
c.1.4	13	c.10.1	3	c.44.2	2	c.72.2	5
c.1.5	1	c.10.2	6	c.45.1	10	c.73.1	3
c.1.6	6	c.13.1	2	c.46.1	12	c.74.1	3
c.1.7	6	c.13.2	3	c.47.1	61	c.76.1	4
c.1.8	80	c.14.1	16	c.48.1	6	c.77.1	6
c.1.9	27	c.15.1	2	c.49.1	2	c.78.1	5
c.1.10	31	c.16.1	2	c.49.2	1	c.78.2	2
c.1.11	11	c.17.1	5	c.50.1	7	c.79.1	6
c.1.12	11	c.18.1	4	c.51.1	3	c.80.1	9
c.1.13	1	c.19.1	2	c.51.2	1	c.81.1	6
c.1.14	3	c.20.1	1	c.51.3	2	c.82.1	7
c.1.15	7	c.21.1	1	c.51.4	7	c.83.1	3
c.1.16	6	c.22.1	1	c.51.5	1	c.84.1	3
c.1.17	2	c.23.1	15	c.51.6	1	c.86.1	1
c.1.18	6	c.23.4	2	c.52.1	26	c.87.1	14
c.1.19	2	c.23.5	13	c.52.2	2	c.88.1	1
c.1.20	1	c.23.6	1	c.52.3	1	c.90.1	2
c.1.21	3	c.23.8	1	c.53.2	2	c.91.1	3
c.1.22	1	c.23.10	9	c.54.1	3	c.92.1	3
c.1.23	2	c.23.11	1	c.55.1	41	c.92.2	9
c.1.25	1	c.23.12	5	c.55.2	2	c.93.1	11
c.1.26	1	c.23.13	1	c.55.3	22	c.94.1	41
c.1.27	1	c.23.14	5	c.55.5	2	c.95.1	15
c.1.28	1	c.23.15	1	c.55.7	2	c.96.1	1
c.1.29	1	c.23.16	22	c.56.2	9	c.97.1	11
c.1.30	1	c.24.1	5	c.56.3	2	c.97.3	1
c.1.31	1	c.25.1	8	c.56.4	1	c.98.1	1
c.2.1	128	c.26.1	20	c.56.5	19	c.98.2	1
c.3.1	41	c.26.2	16	c.56.7	2	c.100.1	2
c.4.1	6	c.26.3	2	c.57.1	6	c.101.1	2
c.5.1	2	c.27.1	1	c.58.1	7	c.103.1	2
c.6.1	3	c.28.1	3	c.59.1	5	c.104.1	2
c.6.2	10	c.30.1	11	c.60.1	5	c.105.1	1
c.6.3	2	c.31.1	16	c.61.1	15	c.106.1	2
c.7.1	3	c.32.1	1	c.62.1	8	c.107.1	1
c.8.1	1	c.33.1	5	c.64.1	1	c.108.1	39
c.8.2	4	c.34.1	2	c.65.1	5	c.109.1	4

Continued on next page

Table S3 – continued from previous page

sf	freq	sf	freq	sf	freq	sf	freq
c.8.3	1	c.36.1	25	c.66.1	63	c.110.1	2
c.8.4	1	c.37.1	121	c.67.1	43	c.111.1	1
c.112.1	1	d.15.9	1	d.52.3	3	d.58.57	3
c.113.1	2	d.15.10	1	d.52.5	1	d.58.58	2
c.114.1	3	d.15.11	1	d.52.7	2	d.58.59	1
c.116.1	9	d.15.12	1	d.52.8	1	d.58.62	1
c.117.1	3	d.16.1	17	d.52.9	1	d.59.1	1
c.119.1	3	d.17.1	6	d.52.10	1	d.60.1	1
c.120.1	6	d.17.2	8	d.54.1	11	d.61.1	2
c.121.1	2	d.17.3	2	d.55.1	1	d.62.1	1
c.122.1	1	d.17.4	45	d.56.1	1	d.63.1	1
c.123.1	3	d.17.5	1	d.58.1	14	d.64.2	3
c.124.1	19	d.17.6	3	d.58.3	5	d.65.1	4
c.125.1	1	d.18.1	4	d.58.4	26	d.66.1	3
c.127.1	1	d.19.1	8	d.58.5	7	d.67.1	2
c.129.1	3	d.20.1	11	d.58.6	1	d.67.3	1
c.130.1	1	d.21.1	10	d.58.7	10	d.67.4	1
c.131.1	2	d.22.1	3	d.58.8	2	d.68.1	1
c.132.1	1	d.23.1	2	d.58.9	3	d.68.2	3
c.133.1	2	d.24.1	5	d.58.10	4	d.68.4	1
c.134.1	1	d.25.1	1	d.58.11	4	d.68.6	3
c.136.1	2	d.26.1	8	d.58.12	1	d.70.1	2
c.138.1	1	d.26.2	1	d.58.14	2	d.72.1	1
c.144.1	1	d.26.3	7	d.58.16	3	d.73.1	2
c.145.1	1	d.31.1	2	d.58.17	4	d.74.1	2
c.150.1	3	d.32.1	24	d.58.18	12	d.74.2	1
c.151.1	1	d.33.1	1	d.58.19	3	d.74.3	1
c.154.1	1	d.34.1	1	d.58.20	1	d.74.4	1
d.1.1	5	d.35.1	1	d.58.22	1	d.75.1	1
d.2.1	12	d.36.1	1	d.58.23	2	d.77.1	1
d.3.1	33	d.37.1	14	d.58.24	2	d.78.1	1
d.4.1	4	d.38.1	40	d.58.25	1	d.79.1	5
d.5.1	4	d.40.1	2	d.58.26	4	d.79.2	3
d.6.1	1	d.41.1	5	d.58.28	2	d.79.3	3
d.8.1	1	d.41.2	2	d.58.29	2	d.79.4	5
d.9.1	7	d.41.5	1	d.58.30	1	d.79.5	2
d.9.2	1	d.42.1	5	d.58.31	3	d.79.6	1
d.12.1	1	d.43.1	1	d.58.32	4	d.79.7	2
d.13.1	9	d.44.1	4	d.58.33	2	d.79.8	1
d.14.1	20	d.45.1	2	d.58.34	2	d.79.9	1
d.15.1	13	d.47.1	1	d.58.36	2	d.80.1	7
d.15.2	5	d.48.1	1	d.58.38	2	d.81.1	15
d.15.3	5	d.50.1	3	d.58.40	2	d.81.2	1
d.15.4	12	d.50.2	1	d.58.48	4	d.81.3	2
d.15.5	1	d.50.3	2	d.58.50	1	d.81.4	1
d.15.6	8	d.51.1	5	d.58.53	2	d.82.1	1

Continued on next page

Table S3 – continued from previous page

sf	freq	sf	freq	sf	freq	sf	freq
d.15.7	2	d.52.1	2	d.58.55	1	d.82.2	2
d.15.8	1	d.52.2	1	d.58.56	2	d.82.5	1
d.83.1	2	d.122.1	9	d.168.1	3	d.227.1	6
d.84.1	1	d.124.1	6	d.169.1	20	d.230.1	1
d.86.1	1	d.126.1	7	d.170.1	2	d.230.2	1
d.87.1	6	d.127.1	6	d.170.2	1	d.231.1	1
d.87.2	4	d.128.1	2	d.171.1	2	d.232.1	1
d.88.1	1	d.129.1	4	d.172.1	1	d.233.1	2
d.89.1	5	d.129.2	1	d.173.1	1	d.235.1	1
d.90.1	8	d.129.3	13	d.174.1	2	d.238.1	1
d.92.1	19	d.129.4	1	d.175.1	1	d.240.1	2
d.92.2	6	d.129.5	1	d.176.1	1	d.241.2	1
d.93.1	9	d.129.7	1	d.177.1	3	d.242.1	1
d.94.1	3	d.129.8	1	d.178.1	2	d.243.1	2
d.95.2	3	d.129.9	1	d.179.1	1	d.246.1	1
d.96.1	9	d.129.11	1	d.180.1	1	d.248.1	1
d.96.2	1	d.130.1	3	d.182.1	1	d.249.1	1
d.97.1	1	d.131.1	14	d.184.1	2	d.250.1	4
d.98.1	1	d.133.1	5	d.185.1	1	d.251.1	1
d.98.2	1	d.134.1	2	d.189.1	4	d.252.1	1
d.100.1	1	d.136.1	7	d.190.1	6	d.254.1	2
d.100.2	1	d.137.1	1	d.192.1	1	d.255.1	1
d.101.1	3	d.139.1	5	d.193.1	1	d.256.1	1
d.103.1	1	d.142.1	14	d.194.1	4	d.257.1	1
d.104.1	9	d.142.2	3	d.196.1	1	d.258.1	2
d.105.1	3	d.143.1	2	d.197.1	1	d.261.1	1
d.106.1	6	d.144.1	31	d.198.1	7	d.264.1	3
d.107.1	4	d.145.1	18	d.198.3	1	d.265.1	7
d.108.1	43	d.146.1	1	d.198.4	1	d.268.1	2
d.109.1	10	d.147.1	1	d.198.5	1	d.269.1	2
d.110.1	2	d.149.1	1	d.199.1	1	d.270.1	1
d.110.2	3	d.150.1	1	d.201.1	1	d.273.1	3
d.110.3	5	d.151.1	8	d.202.1	1	d.274.1	1
d.110.4	1	d.152.1	1	d.207.1	1	d.275.1	1
d.110.5	1	d.153.1	13	d.208.1	1	d.276.1	1
d.110.6	3	d.153.2	1	d.210.1	2	d.278.1	3
d.110.7	4	d.154.1	1	d.211.1	6	d.279.1	1
d.110.8	1	d.155.1	1	d.211.2	1	d.280.1	1
d.111.1	3	d.156.1	2	d.212.1	2	d.281.1	1
d.112.1	2	d.157.1	21	d.213.1	1	d.283.1	2
d.113.1	20	d.159.1	13	d.215.1	1	d.284.1	3
d.114.1	3	d.160.1	2	d.217.1	1	d.286.1	1
d.115.1	4	d.161.1	3	d.218.1	10	d.287.1	1
d.116.1	3	d.162.1	5	d.219.1	2	d.290.1	4
d.117.1	3	d.163.1	1	d.222.1	1	d.291.1	1
d.118.1	5	d.165.1	8	d.223.1	2	d.293.1	1

Continued on next page

Table S3 – continued from previous page

sf	freq	sf	freq	sf	freq	sf	freq
d.120.1	3	d.166.1	11	d.224.1	1	d.296.1	1
d.121.1	1	d.167.1	4	d.226.1	1	d.298.1	1
d.299.1	1	d.380.1	1	f.10.1	1	g.13.1	2
d.300.1	2	d.381.1	1	f.13.1	4	g.14.1	3
d.303.1	1	d.383.1	1	f.14.1	2	g.16.1	2
d.304.1	1	e.1.1	7	f.15.1	1	g.17.1	4
d.306.1	2	e.3.1	22	f.17.2	2	g.18.1	6
d.308.1	2	e.5.1	4	f.17.4	1	g.19.1	1
d.309.1	1	e.6.1	6	f.19.1	1	g.20.1	1
d.310.1	2	e.7.1	6	f.21.1	1	g.21.1	1
d.311.1	1	e.8.1	9	f.21.2	1	g.23.1	1
d.312.1	1	e.10.1	1	f.21.3	1	g.24.1	7
d.313.1	1	e.12.1	1	f.23.1	1	g.27.1	2
d.314.1	1	e.13.1	1	f.23.2	1	g.28.1	1
d.316.1	1	e.15.1	1	f.23.3	1	g.30.1	1
d.319.1	1	e.17.1	4	f.23.4	1	g.32.1	1
d.320.1	1	e.18.1	1	f.23.5	1	g.35.1	4
d.321.1	1	e.19.1	1	f.23.6	1	g.36.1	1
d.322.1	1	e.22.1	7	f.23.7	1	g.37.1	3
d.326.1	3	e.23.1	4	f.23.10	2	g.39.1	11
d.328.1	1	e.24.1	1	f.23.21	1	g.41.1	1
d.329.1	1	e.26.1	3	f.23.22	1	g.41.2	2
d.330.1	1	e.27.1	1	f.24.1	1	g.41.3	1
d.331.1	1	e.38.1	1	f.25.1	1	g.41.4	1
d.338.1	1	e.39.1	1	f.26.1	2	g.41.5	5
d.340.1	1	e.50.1	2	f.40.1	1	g.41.9	1
d.341.1	1	e.51.1	1	f.44.1	1	g.41.11	1
d.342.1	1	e.52.1	1	f.51.1	1	g.41.13	1
d.344.1	3	e.53.1	1	f.54.1	1	g.41.14	1
d.345.1	1	e.54.1	1	f.56.1	1	g.41.16	1
d.347.1	2	e.57.1	1	g.1.1	2	g.44.1	2
d.351.1	1	e.60.1	1	g.3.1	3	g.45.1	2
d.354.1	1	e.62.1	1	g.3.2	2	g.46.1	1
d.356.1	1	e.65.1	1	g.3.7	3	g.49.1	1
d.359.1	1	e.71.1	1	g.3.9	1	g.50.1	4
d.363.1	1	e.72.1	1	g.3.11	16	g.52.1	4
d.365.1	1	e.74.1	1	g.3.13	2	g.68.1	6
d.366.1	1	f.1.2	1	g.3.15	2	g.69.1	1
d.367.1	2	f.1.4	2	g.3.19	1	g.74.1	2
d.369.1	3	f.1.5	1	g.4.1	1	g.75.1	1
d.370.1	1	f.3.1	2	g.7.1	10	g.77.1	1
d.373.1	1	f.4.1	3	g.8.1	4	g.81.1	1
d.376.1	1	f.4.3	5	g.9.1	5	g.86.1	1
d.377.1	1	f.6.1	3	g.10.1	1	g.91.1	1
d.379.1	1	f.7.1	3	g.12.1	1		
